# The Effect of Intervertebral Cartilage on Neutral Posture and Range of Motion in the Necks of Sauropod Dinosaurs

**DOI:** 10.1371/journal.pone.0078214

**Published:** 2013-10-30

**Authors:** Michael P. Taylor, Mathew J. Wedel

**Affiliations:** 1 Department of Earth Sciences, University of Bristol, Bristol, United Kingdom; 2 College of Osteopathic Medicine of the Pacific and College of Podiatric Medicine, Western University of Health Sciences, Pomona, California, United States of America; University of Pennsylvania, United States of America

## Abstract

The necks of sauropod dinosaurs were a key factor in their evolution. The habitual posture and range of motion of these necks has been controversial, and computer-aided studies have argued for an obligatory sub-horizontal pose. However, such studies are compromised by their failure to take into account the important role of intervertebral cartilage. This cartilage takes very different forms in different animals. Mammals and crocodilians have intervertebral discs, while birds have synovial joints in their necks. The form and thickness of cartilage varies significantly even among closely related taxa. We cannot yet tell whether the neck joints of sauropods more closely resembled those of birds or mammals. Inspection of CT scans showed cartilage:bone ratios of 4.5% for *Sauroposeidon* and about 20% and 15% for two juvenile *Apatosaurus* individuals. In extant animals, this ratio varied from 2.59% for the rhea to 24% for a juvenile giraffe. It is not yet possible to disentangle ontogenetic and taxonomic signals, but mammal cartilage is generally three times as thick as that of birds. Our most detailed work, on a turkey, yielded a cartilage:bone ratio of 4.56%. Articular cartilage also added 11% to the length of the turkey's zygapophyseal facets. Simple image manipulation suggests that incorporating 4.56% of neck cartilage into an intervertebral joint of a turkey raises neutral posture by 15°. If this were also true of sauropods, the true neutral pose of the neck would be much higher than has been depicted. An additional 11% of zygapophyseal facet length translates to 11% more range of motion at each joint. More precise quantitative results must await detailed modelling. In summary, including cartilage in our models of sauropod necks shows that they were longer, more elevated and more flexible than previously recognised.

## Introduction

### Historical background

**Figure 1:**
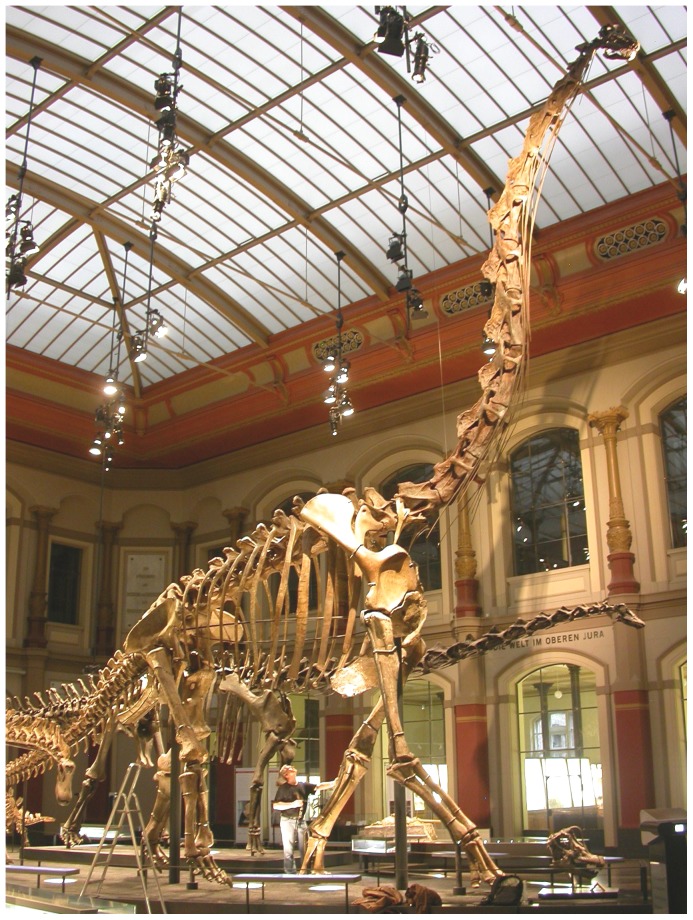
The world's biggest mounted skeleton: the sauropod *Giraffatitan brancai*. Mounted skeleton of *Giraffatitan brancai* paralectotype MB.R.2181 at the Museum für Naturkunde Berlin, Berlin, Germany. Lead author for scale, by the skeleton's elbow. This is the largest mounted skeleton in the world based primarily on real remains rather than sculptures. It is 13.27 m tall, and represents an animal that probably weighed about 20–30 tonnes[61]. Much larger sauropods existed, but they are known only from fragmentary remains.

Sauropod dinosaurs are notable both for their very long necks [1] and their very large body sizes [2] (Figure 1). They were, by an order of magnitude, the heaviest terrestrial animals that have ever existed [3]. An extensive review of sauropod palaeobiology [4] found that the long necks of sauropods were the key factor in the evolution of their large size.

Ever since the sauropod body shape has been understood, the posture and flexibility of their necks has been of interest. Initially, the long neck was assumed to be “swanlike” and flexible [5]–[7], and habitually held high above the level of the torso. Elevated posture was depicted in most (though not all) life restorations of sauropods, including the classic works of Knight [8], Zallinger [9] and Burian [10], and continued to dominate the popular perception of sauropods through books such as *The Dinosaur Heresies* [11] and films such as *Jurassic Park* [12].

This changed in 1999, with the work of Stevens and Parrish [13]. In a short paper, Martin had proposed, based on his work on mounting the skeleton of the Middle Jurassic sauropod *Cetiosaurus*, that it was constrained to a relatively low, horizontal neck posture, and limited in flexibility [14]. Stevens and Parrish extended this idea to the better known Late Jurassic sauropods *Apatosaurus* and *Diplodocus*, and modelled the intervertebral articulations using a computer program of their own devising named DinoMorph. They concluded that *Apatosaurus* and *Diplodocus*, and by extension other sauropods, were adapted to “ground feeding or low browsing” and stated that “*Diplodocus* was barely able to elevate its head above the height of its back”. The horizontal neck postures advocated in this widely publicised paper were quickly adopted as a new orthodoxy, and were reflected in the BBC television documentary *Walking With Dinosaurs* [15] and a special exhibition at the American Museum of Natural History. Stevens [16] subsequently published a high-level description of the DinoMorph software, and Stevens and Parrish [17], [18] elaborated their earlier work with more detailed models.

Although several subsequent publications have provided evidence for a habitually raised neck posture [19]–[21], the only direct response to the work of Stevens and Parrish was that of Upchurch [22], a half-page technical comment. As a result, certain other flaws in this influential study have so far remained unaddressed. This is unfortunate, as the digital modelling approach pioneered by the DinoMorph project is potentially very useful: as a result of the lack of serious critique, this approach has not yet matured into the powerful and informative tool that it should have become.

The year after the DinoMorph work was published, Gregory Paul ([23]: 92–93) pointed out the importance of cartilage in understanding posture:

A problem with estimating neck posture is that it is highly sensitive to the thickness of the cartilage separating the vertebrae, especially the discs. The computer-generated studies [of Stevens and Parrish] have assumed that the discs separating the vertebrae were thin; but so closely spacing the neck vertebrae jams the aft rim of one vertebra's centrum into the base of the rib of the following vertebra in some sauropods. It is therefore probable that at least some sauropods had thick intervertebral discs. The thicker the discs were, the more upwardly flexed the neck was.

But this was rejected by Stevens and Parrish ([18]: 214), as follows:

Paul (2000, 92) suggests that some sauropod necks had thick intervertebral discs, effectively wedged between successive centra, which induced an upward curve at their base. Sauropod necks, however, were strongly opisthocoelous, with central articulations that closely resemble the mammalian opisthocoelous biomechanical design, consisting of condyles that insert deeply in cotyles of matching curvature, leaving little room for cartilage. In modern quadrupeds with opisthocoelous cervicals, such as the horse, giraffe, and rhino, the central condyle and cotyle are separated by only a few millimeters. In avians, heterocoely is similarly associated with very precisely matching articular facets and tight intervertebral separations. Across a large range of extant vertebrates, while substantial intervertebral separations are associated with platycoelous vertebrae, vertebrae with nonplanar central articular geometry generally have little intervening cartilage (pers. obs.), and thus little room for conjecture regarding their undeflected state.

A more general survey of difficulties with the DinoMorph work will be published elsewhere (Taylor and Wedel in prep.) In this contribution, we ignore problems such as the imperfect preservation of the sauropod vertebrae, and investigate in detail the consequences of just one oversimplification: the neglect of articular cartilage in the models used for this work. We show that this significantly affects both the neutral posture recovered and the range of motion found possible.

We examine preserved intervertebral gaps in sauropod necks where CT scans are available, and compare with data obtained from extant animals.

### Basic vertebral architecture

**Figure 2:**
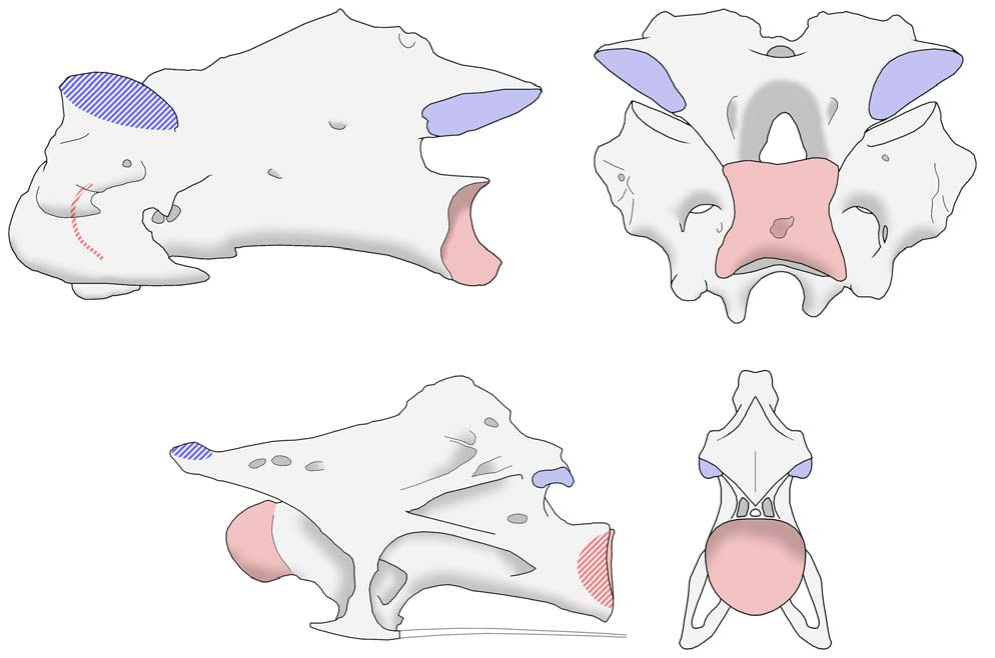
Cervical vertebrae of a turkey and a sauropod. Representative mid-cervical vertebrae from a turkey (top) and the sauropod *Giraffatitan brancai* (bottom), not to scale. Each vertebra is shown in left lateral view (on the left) and posterior view (on the right). Articular surfaces, where each vertebra meets its neighbour, are highlighted in red (for the centra) and blue (for the zygapophyses). Articular surfaces that are concealed from view are cross-hatched: prezygapophyses face upwards and inwards, so that the facets are inclined towards the midline. In sauropods, the centra have ball-and-socket joints. In birds, the joints are saddle-shaped, and the anterior articular surface is hidden in lateral view. Despite numerous differences in detail, the bird and sauropods vertebrae strongly resemble each other in fundamentals.

**Figure 3:**
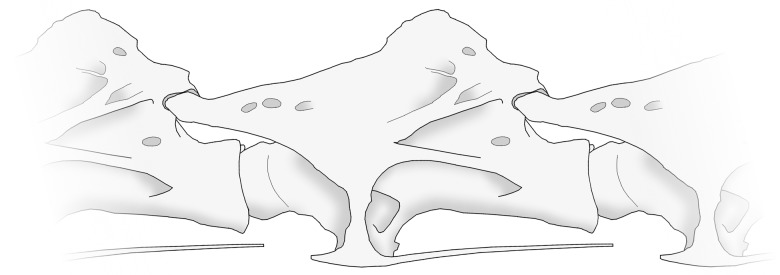
Articulated sauropod vertebrae. Representative mid-cervical vertebra of *Giraffatitan brancai*, articulating with its neighbours. The condyle (ball) on the front of each vertebra's centrum fits into the cotyle (socket) at the back of the preceding one, and the prezygapophyses articulate with the preceding vertebra's postzygapophyses. These vertebrae are in Osteological Neutral Pose, because the pre- and postzygapophyseal facets overlap fully.

The vertebrae of all tetrapods are broadly similar in construction, and those of sauropods and birds particularly resemble each other as a consequence of their close evolutionary relationship (Figure 2). The body of a vertebra is called the centrum, and is usually a fairly simple shape resembling a cylinder. The anterior and posterior facets (i.e., the front and back) of each centrum articulate with the centra of the previous and subsequent vertebrae in the column. Above the centrum is a more elaborate construction called the neural arch. (The neural canal runs from front to back down the middle of the vertebra, between the centrum and arch, and houses the spinal cord.) As well as the centra, adjacent vertebrae also touch at another pair of points above the centra, the zygapophyses. Each vertebra has two pairs of these: prezygapophyses in front and postzygapophyses at the back. Each vertebra's prezygapophyses articulate with the postzygapophyses of the preceding vertebra (Figure 3).

For the purposes of this work, other vertebral features (neural spines, cervical ribs, epipophyses, etc.) are ignored.

### The role and form of intervertebral cartilage

The bone of one vertebra never directly touches the next: instead, the articular surfaces are covered with a thin layer of cartilage, which is softer, smoother and more resilient than bone. Except in rare cases (e.g., [24], [25]), cartilage is not preserved in fossils, and we are unaware of any preserved articular cartilage in sauropod vertebrae. When we speak of fossil vertebrae in this paper, we are referring only to fossilised bone.

**Figure 4:**
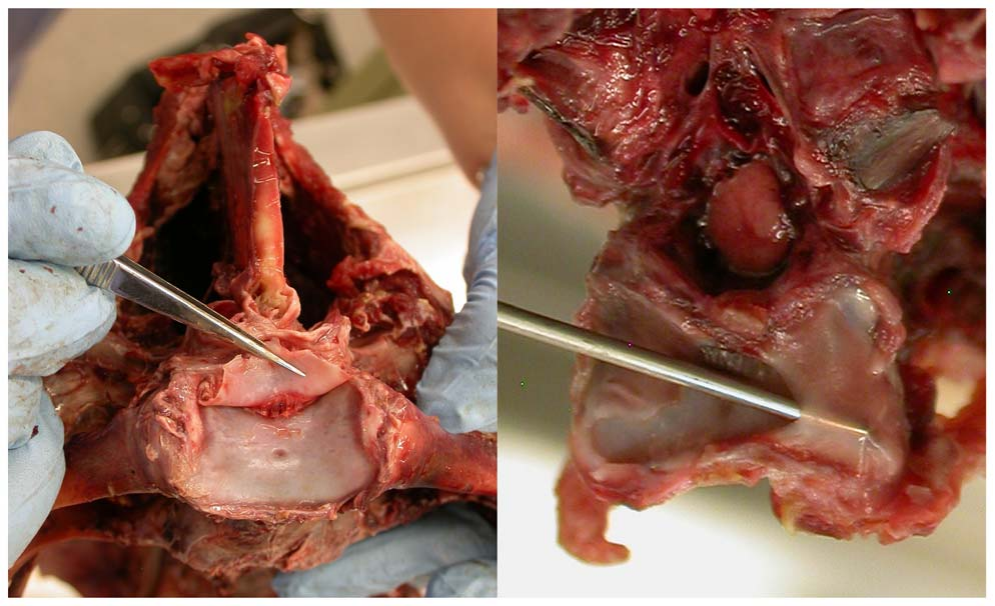
Intervertebral articular discs of an ostrich. Intervertebral articular discs of an ostrich (not to scale). Left: first sacral vertebra in anterior view, showing articular disc of joint with the last thoracic vertebra. Right: posterior view view of a cervical vertebra, with probe inserted behind posterior articular disc. The cervical vertebra is most relevant to the present study, but the the sacral vertebra is also included as it shows the morphology more clearly. These fibrocartilaginous articular discs divide the synovial cavity, like the articular discs in the human temporomandibular and sternoclavicular joints, and should not be confused with the true intervertebral discs of mammals and other animals, which consist of a nucleus pulposus and an annulus fibrosus.

The layers of cartilage covering the articular surfaces of vertebrae do not always closely follow the shape of the underlying bone, but can vary significantly in thickness. For example, the thickness of cartilage between adjacent vertebrae of a king penguin (*Aptenodytes patagonica*) ([26]: figure 4) is more than twice as thick at mid-height as it is at the dorsal and ventral margins. The shape of articular bony surfaces cannot therefore be assumed to indicate the functional shape of those surfaces in life. This is probably true of tetrapods in general but it is particularly important for large non-avian dinosaurs, in which extensive cartilage was present at many joints and did not always reflect the morphology of the underlying bones ([25], [27], [28] but see also [29]).

The morphology of cartilage in intervertebral joints varies significantly among taxa. In most animals, there is a distinct fibrocartilaginous element, known as a disc, between the centra of consecutive vertebrae. These discs consist of an *annulus fibrosus* (fibrous ring), made of several layers of fibrocartilage, surrounding a *nucleus pulposus* (pulpy centre) with the consistency of jelly [30], [31]. But in birds, uniquely among extant animals, there is no separate cartilaginous element. Instead, the articular surfaces of the bones are covered with layers of hyaline cartilage which articulate directly with one another, and are free to slide across each other. The adjacent articular surfaces are enclosed in synovial capsules similar to those that enclose the zygapophyseal joints [32].

The difference between these two constructions is very apparent in dissection: in birds, adjacent vertebrae come apart easily once the surrounding soft tissue is removed; but in mammals, it is very difficult to separate consecutive vertebrae, as they are firmly attached to the intervening intervertebral disc.

Crucially, the extant phylogenetic bracket (EPB) [33] does not help us to establish the nature of the intervertebral articulations in sauropods, as the two extant groups most closely related to them have different articulations. As noted, birds have synovial joints; but crocodilians, like mammals, have fibrocartilaginous intervertebral discs.

To complicate matters further, thin articular discs occur in the necks of some birds – for example, the ostrich (*Struthio camelus*) (Figure 4), the swan (*Cygnus atratus*) ([34]: figure 3), and the king penguin ([26]: figure 4). But these discs do not occur in all birds – for example, they are absent in the turkey (*Meleagris gallopavo*) and the rhea (*Rhea americana*). When they are present, these articular discs divide the synovial cavity and prevent the (cartilage-covered) bones on either side from ever articulating directly with each other, just like the articular discs in the human temporomandibular and sternoclavicular joints. These discs are thinner than the true intervertebral discs of mammals and crocodilians; and they are different in composition, lacking the annulus/nucleus structure and consisting of a simple sheet of fibrocartilage.

**Figure 5:**
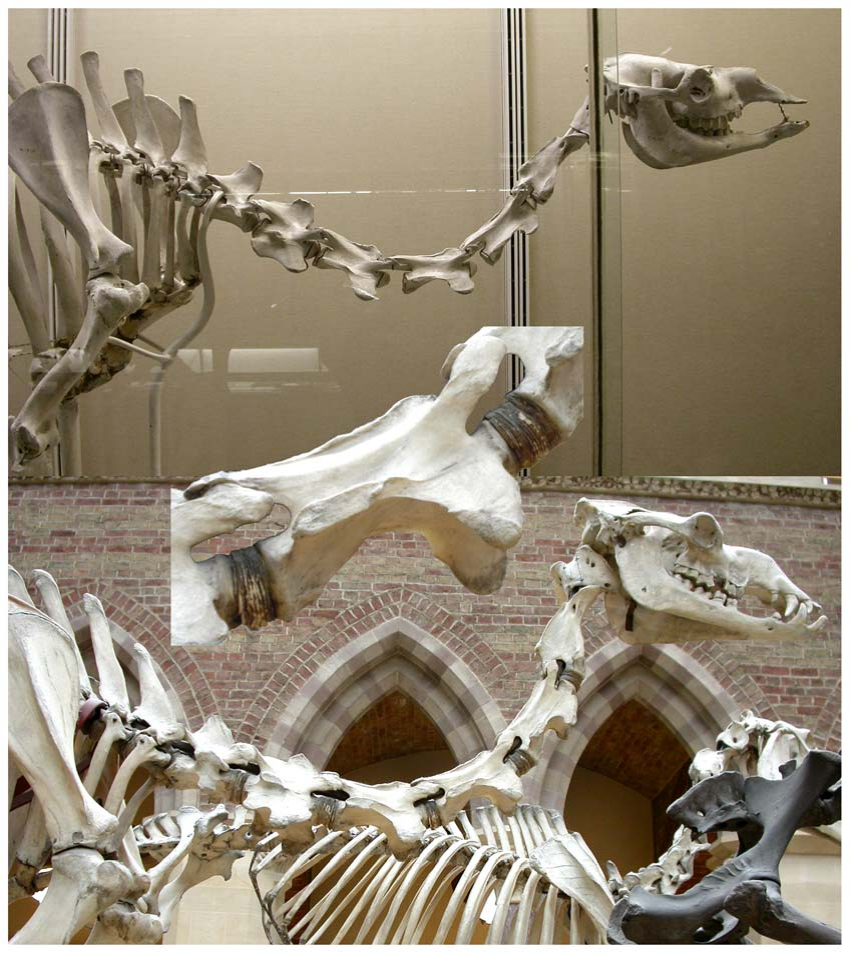
Intervertebral gaps in camel necks. Head and neck of dromedary camels. Top: UMZC H.14191, in right lateral view, posed well below habitual posture, with apparently disarticulated C3/C4 and C4/C5 joints. Photograph taken of a public exhibit at University Museum of Zoology, Cambridge, UK. Bottom: OUMNH 17427, in left lateral view, reversed for consistency with Cambridge specimen. Photograph taken of a public exhibit at Oxford University Museum of Natural History, UK. Inset: detail of C4 of the Oxford specimen, showing articulations with C3 and C5. The centra are separated by thick pads of artificial “cartilage” to preserve spacing as in life.

The thickness of cartilage between consecutive cervical vertebrae is considerable in at least some taxa. For example, in the dromedary camel (*Camelus dromedarius*), mounted skeletons that omit spacers where the cartilage would have been in life instead have large gaps between the centra, even when the neck is posed well below habitual posture (Figure 5).

In this paper, we express thickness of cartilage as a cartilage/bone percentage. This is not to be confused with the percentage of *total* segment length that is accounted for by cartilage: when a 10 cm bone has 1 cm of cartilage on the end, the cartilage/bone ratio is 10%, but cartilage accounts for only 9.09% – one eleventh – of the total segment length.

### Osteological neutral pose (ONP) and range of motion (ROM)

Stevens and Parrish [13] introduced the notion of Osteological Neutral Pose (ONP), which is attained when the centra abut without gaps and the zygapophyseal facets of consecutive vertebrae are maximally overlapped. The vertebrae in Figure 3 are in ONP.

**Figure 6:**
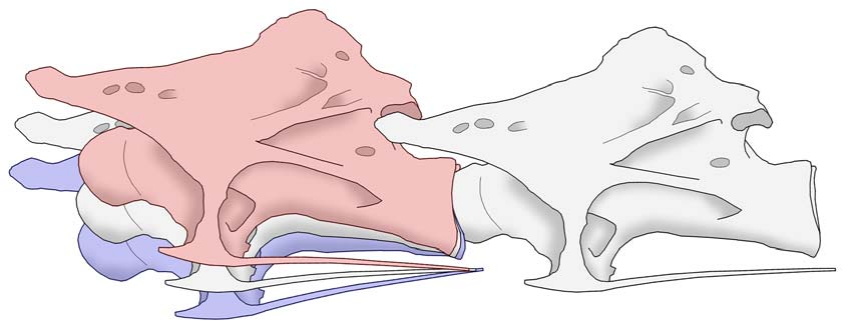
Range of motion in a vertebral joint. Range of Motion (ROM) illustrated schematically for a single intervertebral joint of *Giraffatitan brancai*. The grey-scale vertebrae are shown in Osteological Neutral Pose. The red vertebra has been rotated upwards (“extended”) until its postzygapophyseal facet overlaps 50% with the prezygapophyseal facet of the succeeding vertebra, in accordance with the assumption of Stevens and Parrish. Similarly, the blue vertebra has been rotated downwards (“flexed”) until 50% zygapophyseal overlap is achieved. Because the zygapophyseal articulations in the neck of *Giraffatitan* are some way anterior to the those of the centra, the relative movement of the articulating zygapophyseal facets is anteroventral–posterodorsal; in taxa such as the turkey in which the zygapophyseal articulation are directly above those of the centra, relative movement is anterior-posterior.

When the neck extends or flexes (bends upwards or downwards respectively) the centra remain in articulation, rotating against each other, and the zygapophyses glide past each other. The point around which a pair of consecutive centra rotate with respect to one another is called their centre of rotation. Various factors limit how far a given intervertebral joint can rotate: in the extreme case, bone collides with bone, creating an osteological stop. More often, rotation is inhibited before this point is reached by limits to zygapophyseal travel. The joint between one vertebra's postzygapophysis and the prezygapophysis of the next is enclosed in a delicate synovial capsule which cannot be stretched indefinitely. Stevens and Parrish stated that “pre- and postzygapophyses could only be displaced to the point where the margin of one facet reaches roughly the midpoint of the other facet” [13], citing unpublished data. Range Of Motion (ROM) in their sense is the degree of movement that can be attained while retaining at least 50% overlap between zygapophyseal facets (Figure 6). Although this figure remains to be demonstrated, and is in fact contradicted by Stevens and Parrish themselves ([17]: 191), who observed that when giraffes bend their necks laterally there is almost no zygapophyseal overlap, we provisionally accept the 50% overlap criterion here.

For the purposes of this discussion, ROM is considerably simplified from the reality. The shapes of zygapophyseal facets can be complex, and limit or facilitate motion. The inclination of facets introduces further complexity. As shown in Figure 6, anterior positioning of the zygapophyses in some sauropods (unlike the situation in birds) means that zygapophyseal displacement is primarily dorsoventral rather than anteroposterior. In some cases, zygapophyseal facets can pull apart rather than remaining in articulation. As a final simplification, in this paper we consider only vertical movement of the neck, not lateral movement or twisting. Despite these simplifications, ROM remains a useful abstraction, and its relation to zygapophyseal facet size is apparent: ROM varies more or less linearly with facet size and inversely with distance from zygapophyses to the centre of rotation. Equal ranges of motion can be achieved by small zygapophyseal facets close to the centre of rotation, or larger facets further from it.

## Materials and Methods

### Extinct animal specimens

OMNH 53062 is the holotype of the long-necked basal titanosauriform *Sauroposeidon*. The specimen consists of four articulated mid-cervical vertebrae. Portions of the three more anterior vertebrae were CT scanned in January 1998 to image their pneumatic internal structures [35]–[37]. This is the first time that these scans have been used to investigate the shapes of the articular surfaces of the vertebrae or to estimate the thickness of the intervertebral cartilage.

CM 3390 and CM 11339 are two partial skeletons of juvenile individuals of *Apatosaurus*. They were collected from the Carnegie Museum Quarry at Dinosaur National Monument, which also yielded CM 3018, the holotype of *Apatosaurus louisae*. To date, no single quarry has produced members of more than one valid species of *Apatosaurus*, and according to McIntosh ([38]: 26) these specimens “show no characters to distinguish them from the above [holotype] specimens of *Apatosaurus louisae*.” For the purposes of this discussion, we accept this tentative referral.

### Extant animal specimens

It is impossible to fully determine the effect of articular cartilage on ONP and ROM of sauropod necks directly due to the paucity of specimens with preserved cartilage. As a proxy, we took measurements from the neck of a domestic turkey, sourced from a local butcher. We interpreted these as proportions of whole-neck length, vertebra length and zygapophysis length.

Turkeys are a reasonable model organism for these purposes, as birds are the closest living relatives of sauropods and their cervical architecture is similar [1], [39], but see the discussion below of other animals' necks that are used as well.

The complete neck of the turkey is made up of 14 vertebrae [40], of which the last few are functionally part of the torso. However, the neck obtained for this work is incomplete, consisting of only eight vertebrae. Based on the absence of carotid processes in the most posterior vertebra, this is probably C13, meaning that the available neck segments represent C6–C13. This is consistent with the profiles of the vertebrae illustrated by Harvey et al. ([40]: plate 65). Although the absence of the first five vertebrae is regrettable, it is not critical as the base of the neck is the region where flexion and extension have the greatest effect on posture.

We also obtained less detailed cartilage measurements for a selection of other extant animals as detailed below. The ostrich, rhea, alligator (*Alligator mississippiensis*) and horse (*Equus caballus*) are all salvage specimens, and they were obtained, dissected, and photographed with the approval of the Institutional Animal Care and Use Committee at Western University of Health Sciences. The camel is a mounted museum specimen, the dog is a veterinary subject, and the giraffe was obtained from an anonymous zoo via the Royal Veterinary College, UK.

We are all too aware that the wildly different provenances and ages of these specimens, and the different measurement techniques used, make direct comparisons problematic. As noted in the Future Work section below, we hope subsequent studies will be able to take advantage of a wider and more controlled range of specimens.

### Fossil CT scanning protocol

Sauropod vertebrae were CT scanned at the University of Oklahoma Medical Center in Oklahoma City in January 1998 (*Sauroposeidon*) and January 2000 (both specimens of *Apatosaurus*). CT scans were performed using a General Electric 9800 Highlight Advantage 4th generation scanner. Scout images were obtained in lateral projection with a technique setting of 120 kVp (kilovolt peak) and 40 mA (milliamperes). Axial images were produced at 120 kVp and 120 mA. Data were reconstructed in bone algorithm using a Star Tech, Inc., One Sun CPU computed tomography array imaging processor and the GE Advantage version 1.0 imaging software package.

### Vertebra measurement protocol

In order to determine the thickness of intervertebral cartilage and possible other soft-tissue, it is necessary to accurately measure the length of both intact neck segments and their constituent vertebrae.

Measuring the lengths of intact necks is awkward, even when the heads and torsos have been removed. Contraction of dorsal tension members causes them to curl up, which impedes attempts to find the straight-line length. It is necessary to hold a neck straight, and simultaneously to gently compress it end-to-end in order to prevent artificial elongation due to post-mortem separation of adjacent vertebrae. This is hard to achieve without buckling the neck out of the straight line. With the neck straightened and longitudinally compressed, a measurement must be taken along the neck, between perpendiculars, from the front of the anteriormost vertebra to the back of the posteriormost.

**Figure 7:**
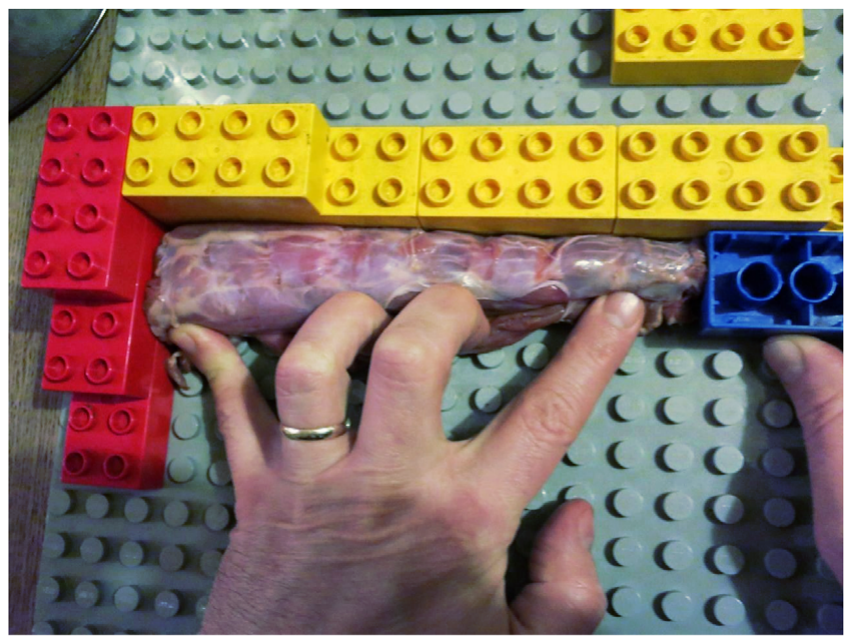
Measurement rig for necks. Measurement rig for intact turkey necks, constructed from Duplo bricks and baseboard. The neck is pushed into the angle between the back wall (yellow) and the left wall (red), and held straight along the back wall. The marker brick (blue) abuts the end of the neck: the distance between the left wall and the marker brick is the length of the neck between perpendiculars.

To solve this problem, a simple measurement rig was constructed from Duplo bricks and a baseboard. The bricks were used to construct an 'L'-shaped bracket (Figure 7). The neck is then laid in this bracket with its dorsal side facing away and into the back wall. It is unrolled and straightened against that wall. Once the neck is in place, with its posterior end hard against the left wall, a marker brick is used to locate the position of the anteriormost part of the neck, sliding along the back wall until the neck prevents further travel. If this is done correctly, there is very little movement: the entire series of vertebrae is lined up and solidly abutted, with bone pushing against the left wall and the marker brick. The distance between left wall and this brick is then the length of the neck. It is easy to remove the neck (without moving the marker brick) and measure this distance.

**Figure 8:**
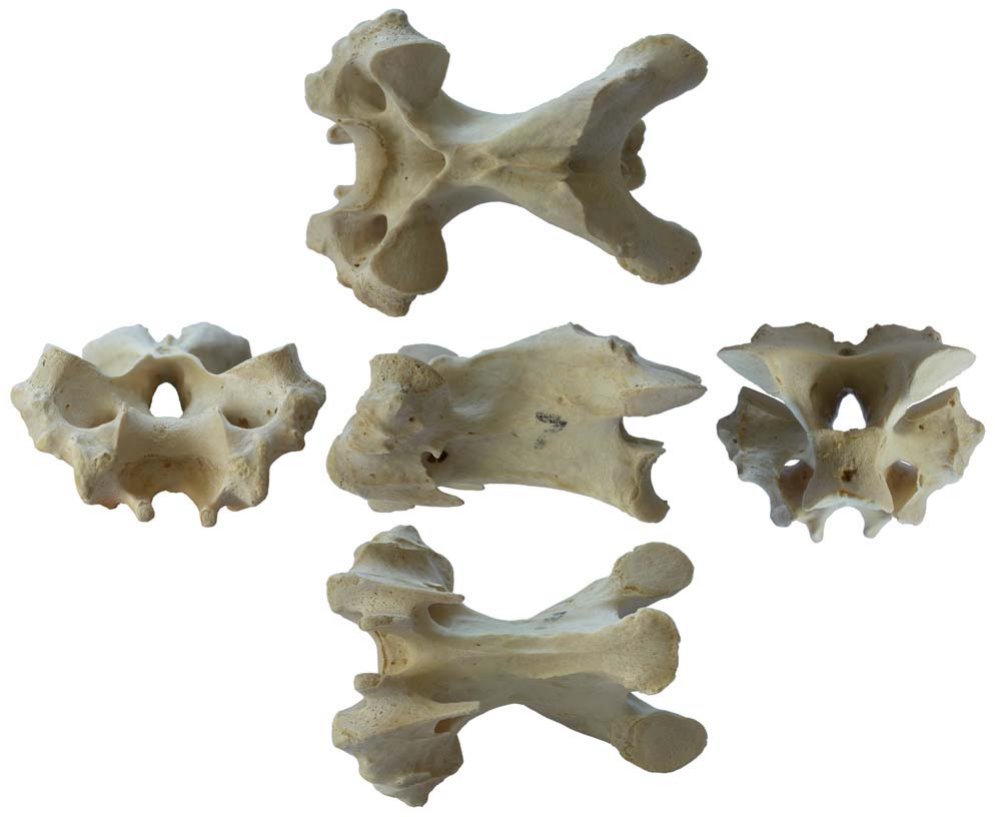
Cervical vertebra 7 from a turkey. Cervical vertebra 7 from a turkey: anterior view on the left; dorsal, left lateral and ventral views in the middle row; and posterior on the right.

Measuring the length of individual cervical vertebrae is also problematic, due to the complex saddle shape (“heterocoely”) of the articular faces of the centrum (Figure 8). The anterior articular surface is convex dorsoventrally but concave transversely, and is not the most anterior part of the vertebra; and the posterior face is concave dorsoventrally and convex transversely. For our purposes, the most interesting metric is not total length (which would include the anteriorly projecting cervical-rib loops and in some cases overhanging postzygapophyses) but functional length.

**Figure 9:**
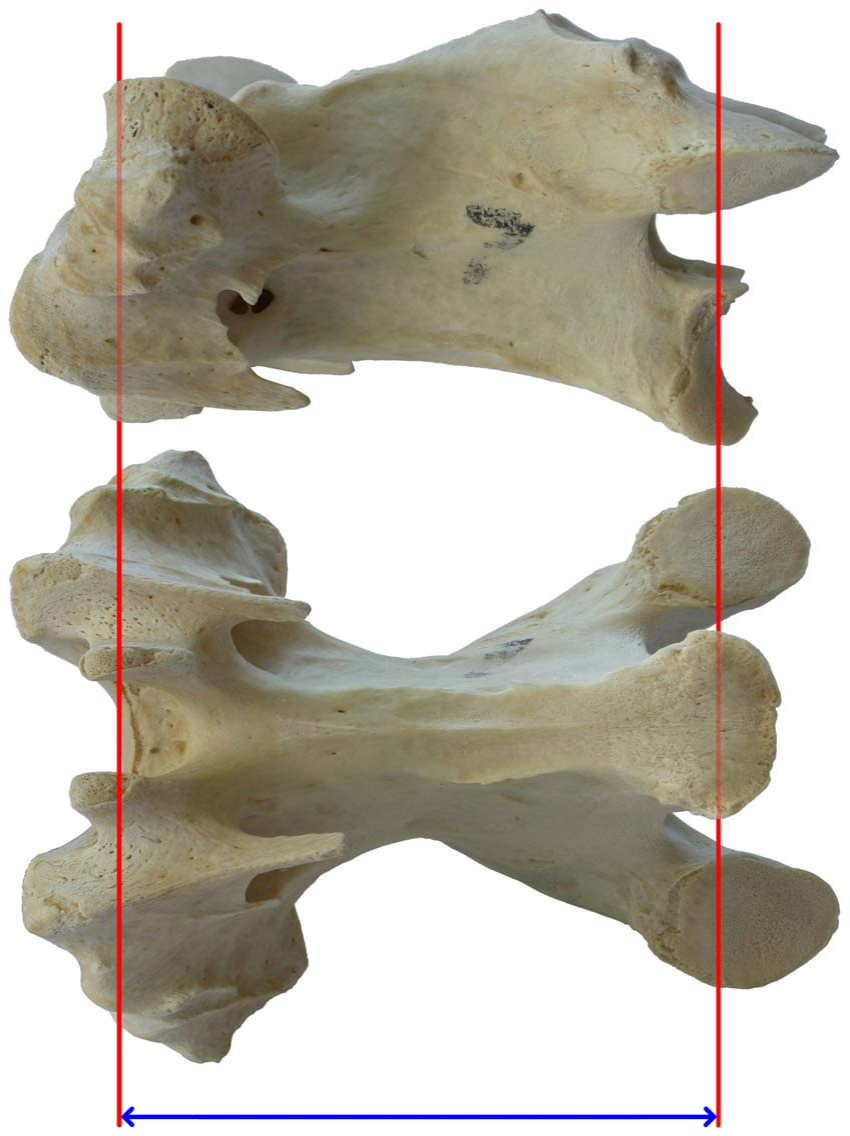
Functional length of a cervical vertebra. Functional centrum length of a cervical vertebra of a turkey. The measurement is taken between the inflection points of the saddle-shaped articulations at each end of the centrum, shown here by the blue arrow connecting the red lines that mark the position of the saddle points.

We define *functional length* as the straight-line distance between the most anterior point on the midline of the anterior face, and the most anterior point on the midline of the posterior face – for birds, that is between the saddle points of the anterior and posterior articular surfaces of the centrum (Figure 9). Functional length can also be thought of as the distance between the same point on two consecutive vertebrae when they are articulated. This definition works for vertebrae of any shape – for example, those of sauropods, which have ball-and-socket joints rather than saddle-shaped joints, also have a functional length equal to the distance between the most anterior points on the midlines of the anterior and posterior faces. Functional length may be measured either including or excluding articular cartilage. We use it exclusive of cartilage except where otherwise noted.

We use functional, rather than total, length because it has the important property that the sum of the functional lengths of a sequence of vertebrae is equal to the functional length of the sequence as a whole.

**Figure 10:**
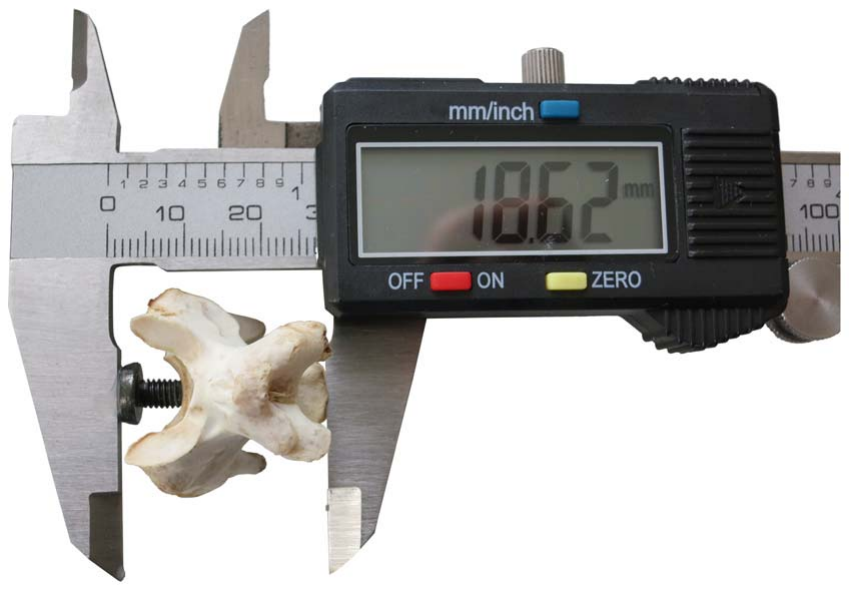
Modified calipers for measuring functional vertebral length. Modified calipers used to measure functional length of a turkey vertebra. The tooth glued to the left jaw protrudes into the transverse concavity of the anterior articular surface and the dorsoventral concavity of the posterior articular surface straddles the right jaw.

To measure the functional length of the turkey vertebrae, we glued a tooth onto one jaw of the calipers, facing the other jaw, and recalibrated them so that they read zero when the tooth was in contact with the opposing jaw. Then we placed the vertebra between the jaws of these modified calipers, with the tooth protruding into the transverse concavity of the anterior articular surface of the centrum, and with the dorsoventral concavity of the posterior articular surface straddling the unmodified jaw (Figure 10).

We also measured the anteroposterior length of all four zygapophyseal facets of each vertebra with unmodified calipers.

Each measurement (functional centrum length and four zygapophyseal facet lengths) was made three times: once on the freshly dissected-out vertebrae; once after they had been simmered and cleaned, and cartilage had been removed from the articular surfaces; and once more after being degreased in dilute hydrogen peroxide and thoroughly dried. The bones of living animals most closely resemble the first of these measurements, while fossil bones most closely resemble the last. The differences between these sets of measurements show how calculations based on fossils mislead as to the behaviour of bones in living animals.

## Results

### Data from sauropod CT scans

#### Sauroposeidon OMNH 53062

**Figure 11:**
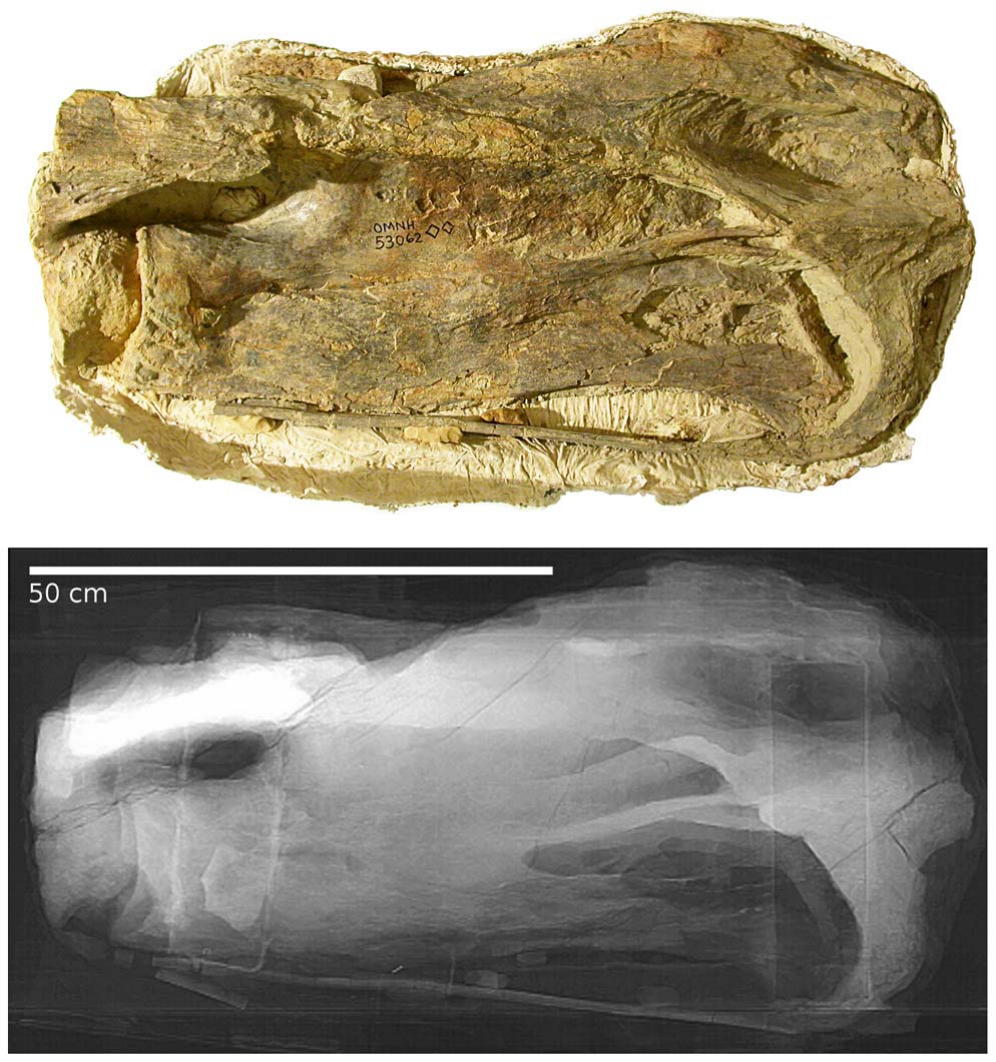
Fifth and partial sixth cervical vertebrae of *Sauroposeidon*. Photograph and x-ray scout image of C5 and the anterior portion of C6 of *Sauroposeidon* OMNH 53062 in right lateral view. The anterior third of C5 eroded away before the vertebra was collected. C6 was deliberately cut through in the field to break the multi-meter specimen into manageable pieces for jacketing (see [37] for details). Note that the silhouettes of the cotyle of C5 and the condyle of C6 are visible in the x-ray.

**Figure 12:**
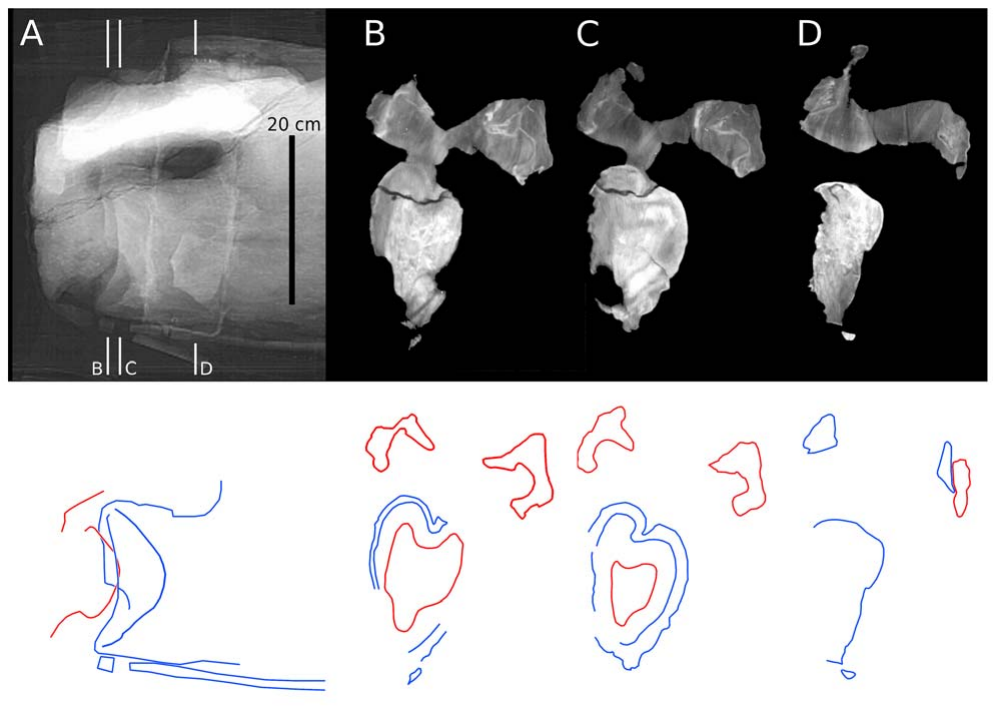
CT slices from fifth cervical vertebrae of *Sauroposeidon*. X-ray scout image and three posterior-view CT slices through the C5/C6 intervertebral joint in *Sauroposeidon* OMNH 53062. In the bottom half of figure, structures from C6 are traced in red and those from C5 are traced in blue. Note that the condyle of C6 is centered in the cotyle of C5 and that the right zygapophyses are in articulation.

The four vertebrae that make up the holotype of *Sauroposeidon* are inferred to represent C5–C8 [35], [36], and we refer to them as such here. The specimen therefore includes three intervertebral joints: between C5 and C6, between C6 and C7, and between C7 and C8. C7 and C8 are simply too large to pass through a medical CT scanner, but the other two joints have been imaged. At the C5/C6 joint, the condyle of C6 is centered in the cotyle of C5, and the zygapophyses on the right are in articulation (Figures 11 and 12). (The left sides of the vertebrae were facing up in the field and were badly damaged by erosion prior to excavation.) As in *Apatosaurus* CM 3390, the cotyle is more rounded than the condyle, so the radial spacing between the vertebrae varies from the rim of the cotyle to the centre. The spacing from the front of the condyle of C6 to the deepest point in the cotyle of C5 is 52 mm, but the minimum radial spacing between the condyle and the cotyle rim is only 31 mm.

**Figure 13:**
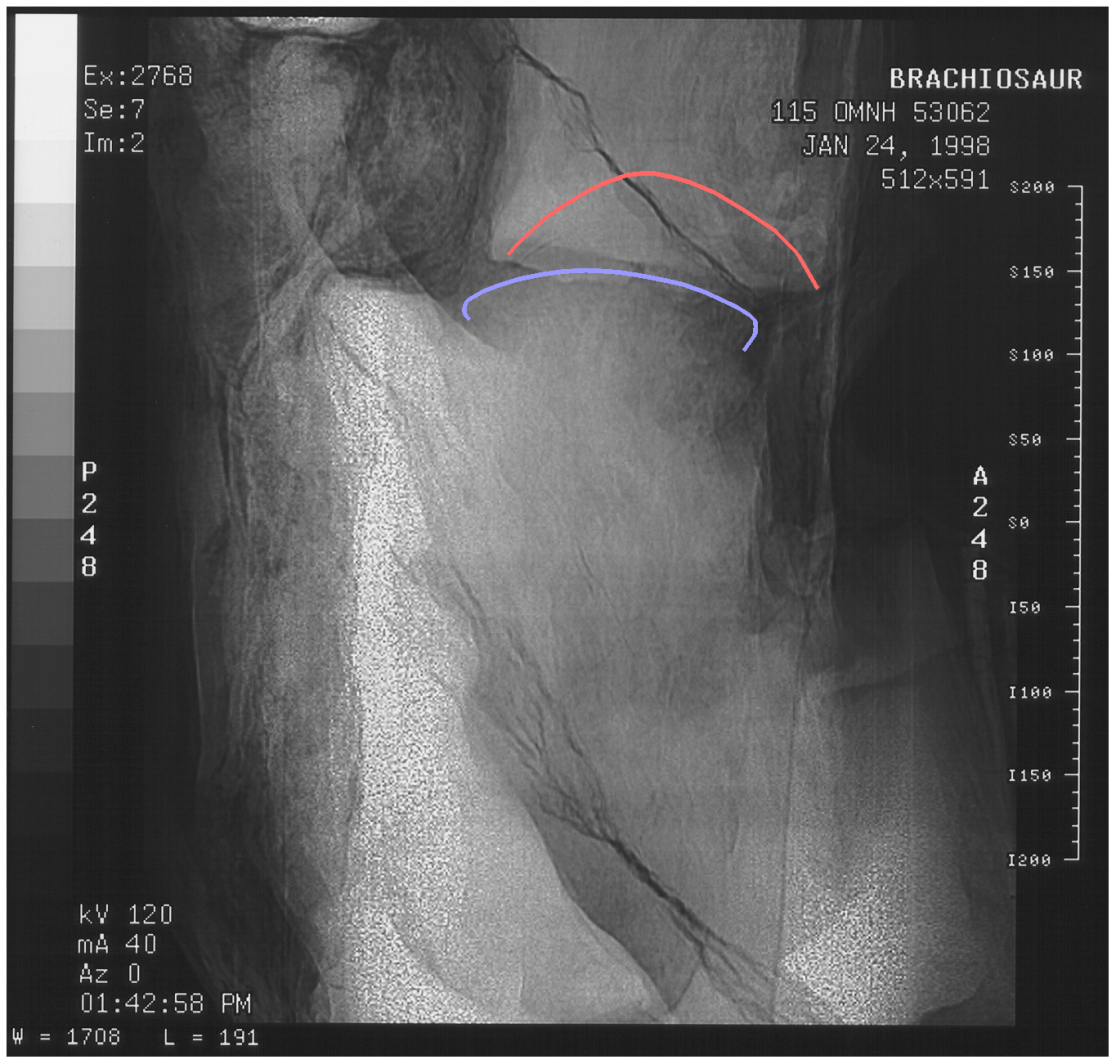
Joint between sixth and seventh cervicals vertebrae of *Sauroposeidon*. X-ray scout image of the C6/C7 intervertebral joint in *Sauroposeidon* OMNH 53062, in right lateral view. The silhouette of the condyle is traced in blue and the cotyle in red. The scale on the right is marked off in centimeters, although the numbers next to each mark are in millimeters.

C6 is slightly flexed relative to C7, and the condyle of C7 is displaced toward the top of the cotyle of C6, rather than being maximally engaged like the C5/C6 joint. The condyle of C7 has a very odd shape. Although the condyle has a maximum dorsoventral diameter of just over 170 mm, it is only about 30 mm long (Figure 13). The unusually flattened shape cannot be an artefact of preparation or damage because the anterior end of the condyle is covered by matrix and surrounded by the cotyle. It is difficult to imagine a form of taphonomic distortion that would act only on the vertebral condyle, and the rest of the vertebrae are anything but anteroposteriorly compressed. Although it looks odd, the condyle of C7 is consistent with the condyle of C6 and with that of D2 in CM 3390 in having a broader, flatter curvature than the cotyle with which it articulated. Assuming a minimum 30 mm radial spacing around the rim of the cotyle, as at the C5/C6 joint, gives a maximum anteroposterior spacing at the centre of about 60 mm.

**Figure 14:**
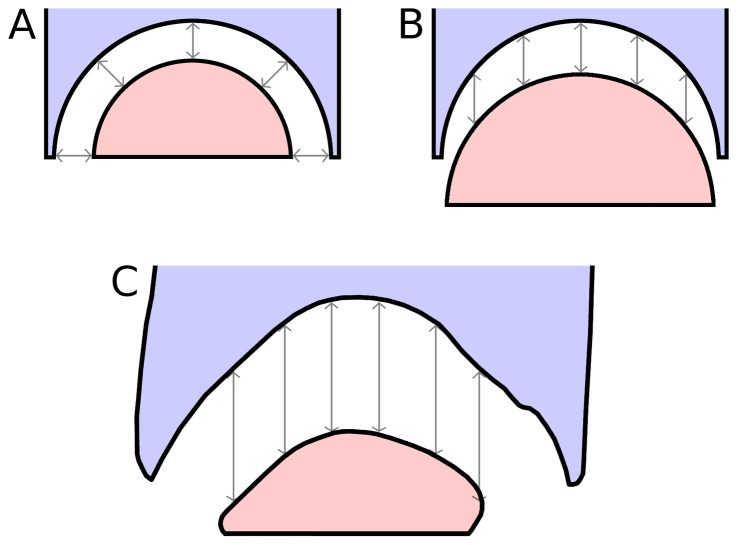
Geometry of opisthocoelous intervertebral joints. Hypothetical models of the geometry of an opisthocoelous intervertebral joint compared with the actual morphology of the C5/C6 joint in *Sauroposeidon* OMNH 53062. A. Model in which the condyle and cotyle are concentric and the radial thickness of the intervertebral cartilage is constant. B. Model in which the condyle and cotyle have the same geometry, but the condyle is displaced posteriorly so the anteroposterior thickness of the intervertebral cartilage is constant. C. the C5/C6 joint in *Sauroposeidon* in right lateral view, traced from the x-ray scout image (see Figure 12); dorsal is to the left. Except for one area in the ventral half of the cotyle, the anteroposterior separation between the C5 cotyle and C6 condyle is remarkably uniform. All of the arrows in part C are 52 mm long.

**Figure 17:**
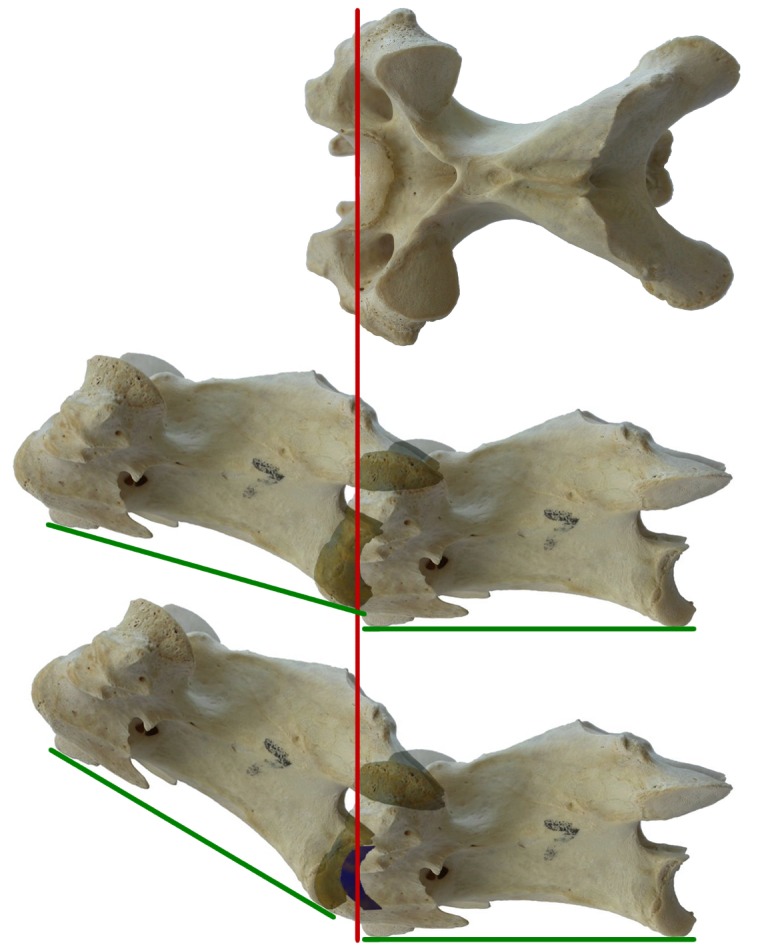
Effect on neutral pose of including cartilage on ONP. Effect on neutral pose of including cartilage. Top: dorsal view of a turkey cervical vertebra: vertical red line indicates the position of the most anterior part of the midline of the anterior articular surface, which is obscured in later view. Second row: two such vertebrae arranged in osteological neutral pose, with the articular surfaces of the centra abutting and the zygapophyseal facets maximally overlapped. The anterior vertebra is inclined by about 16° relative to the posterior. Third row: two such vertebra, with the centrum of the more posterior one elongated by 6.46% to allow for intervertebral cartilage (shown in blue), and the more anterior positioned with its centrum articulating with the cartilage and the zygapophyses maximally overlapped. The anterior vertebra is inclined by about 31°. The inclusion of cartilage has raised neutral posture by 15°. Green lines represent a horizontal baseline, joining the most ventral parts of the anterior and posterior ends of the vertebrae.

Conceptually, we might expect cartilage in a ball-and-socket joint to approach one of two simple conditions: a constant radial thickness, or a constant anteroposterior thickness (Figure 14: parts A and B). Note that in these simple models the condyle is assumed to have the same basic shape as the cotyle. At the two intervertebral joints in *Sauroposeidon* that have been imaged, this expectation is not met – in both cases, the cotyle is deeper and more strongly curved than the condyle. However, at the C5/C6 joint the anteroposterior separation between the condyle and cotyle is almost constant, at least in the sagittal plane (Figure 14: part C). But this even separation is achieved by having a condyle that is much smaller in diameter than the cotyle, and of a different shape. The condyle of C6 is not as flattened as the condyle of C7, but it is still much flatter than the condyles in cervicals of *Giraffatitan* ([41]: figures 17–46) and North American cervicals referred to *Brachiosaurus* ([42]: figure 7.2). It is tempting to speculate that the flattened condyles and nearly constant thickness of the intervertebral cartilage are adaptations to bearing weight, which must have been an important consideration in a cervical series more than 11 meters long, no matter how lightly built.

The cotyles of C5 and C6 are both 65–70 mm deep. So the distance from the foremost point of the C6 condyle to the deepest point of its cotyle includes the centrum length (1220 mm) minus the depth of the C6 cotyle (67 mm), for a total of about 1153 mm from cotyle to cotyle. The maximum cartilage thickness of 52 mm therefore accounts for 4.5% of the bone length, which is proportionally thinner than in most of the other animals we have sampled.

Centrum shape is conventionally quantified by Elongation Index (EI), which is defined as the total centrum length divided by the dorsoventral height of the posterior articular surface. *Sauroposeidon* has proportionally very long vertebrae: the EI of C6 is 6.1. If instead it were 3, as in the mid-cervicals of *Apatosaurus*, the centrum length would be 600 mm. That 600 mm minus 67 mm for the cotyle would give a functional length of 533 mm, not 1153, and 52 mm of cartilage would account for 9.8% of the length of that segment. And, of course, not all of the cervicals in *Sauroposeidon* were so long. Assuming a cervical count of thirteen, multiplying by an average of 52 mm of cartilage per segment comes to 67 cm of cartilage in the neck. Assuming a summed vertebral length of 11.5 meters (based on comparisons with *Brachiosaurus* and *Giraffatitan* [36]), the neck in life would have been just over 12 meters long, for a cartilage/bone ratio of 5–6%.

### Apatosaurus louisae CM 3390

**Figure 15:**
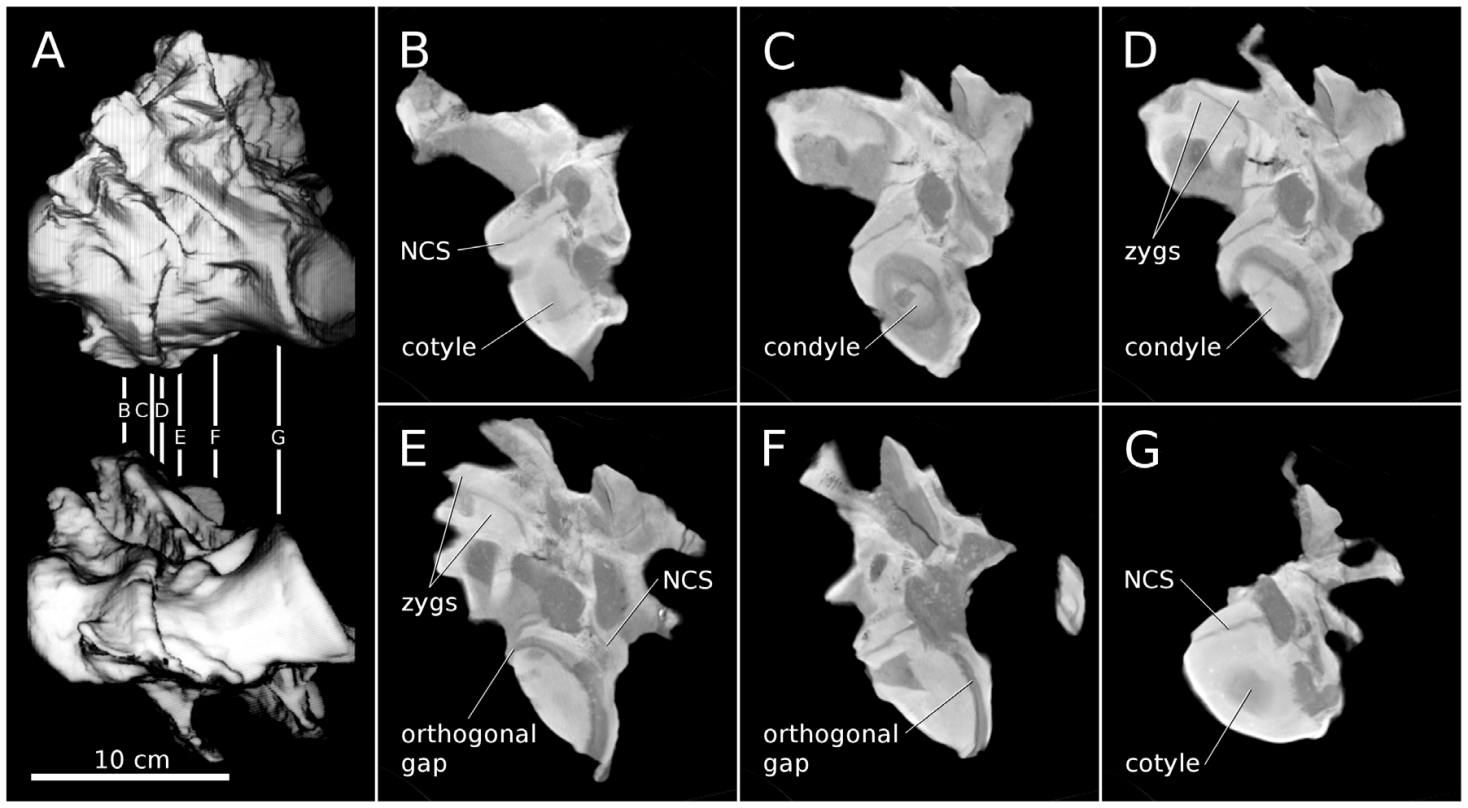
First and second dorsal vertebrae of *Apatosaurus* CM 3390. Articulated first and second dorsal vertebrae of *Apatosaurus* CM 3390. A. Digital model showing the two vertebrae in articulation, in left lateral (top) and ventral (bottom) views. B-G. Representative slices illustrating the cross-sectional anatomy of the specimen, all in posterior view. B. Slice 25. C. Slice 31. D. Slice 33. E. Slice 37. F. Slice 46. G. Slice 61. Orthogonal gaps are highlighted where the margins of the condyle and cotyle are parallel to each other and at right angles to the plane of the CT slice. 'Zygs' is short for 'zygapophyses', and NCS denotes the neurocentral synchondroses.

CM 3390 includes a pair of articulated anterior dorsal vertebrae (Figure 15). The vertebrae lack hyposphenes, as expected for anterior dorsals of *Apatosaurus* ([43]: 201), and based on the centrum proportions and the low positions of the parapophyses on the centra (Figure 15 part A), the vertebrae probably represent the first two dorsals – rather than posterior cervicals, as posited by Wedel ([44]: 349 and figure 7). D2 has a centrum length of 90 mm, a cotyle height of 58 mm, and so an EI of about 1.5. The equivalent vertebra in the mounted holotype of *A. louisae*, CM 3018, has a cotyle height of 225 mm, about 3.9 times the linear size of CM 3390.

The slice thickness in the CT scan is 3 mm, with 1 mm of overlap on either side, yielding a distance of 2 mm from the centre of one slice to the next. Resolution within each slice is 0.571 mm/pixel (44.5 dpi). In this and all other scans, the slices are numbered from anterior to posterior.

The deepest part of the cotyle of D1 is first visible in slice 25 (Figure 15 part B). The condyle of D2 is first apparent in slice 31 (Figure 15 part C). However, we cannot tell where in the 2 mm thickness represented by slice 25 the cotyle actually begins, and the same uncertainty applies to the most anterior point of the condyle within slice 31. The spacing between the vertebrae is therefore at least five slices (26–30) and no more than 7 (25–31, inclusive), or 10–14 mm. The first clear slice through the cotyle of D2 is in slice 61 (Figure 15 part G). So the functional length of D2, measured from the foremost part of the condyle to the deepest part of the cotyle is 29–31 slices or 58–62 mm. The gap for cartilage accounts for 12±2/60±2, a cartilage/bone ratio of 20±4%.

Juvenile sauropods have proportionally short cervicals ([36]: 368–369, figure 14, and table 4). The scanned vertebrae are anterior dorsals with an EI of about 1.5. Mid-cervical vertebrae of this specimen would have EIs about 2, so the same thickness of cartilage would yield a cartilage/bone ratio of 12±2/80±2 or 15±3%. Over ontogeny the mid-cervicals telescoped to achieve EIs of 2.3–3.3. The same thickness of cartilage would then yield a cartilage/bone ratio of 9–13%, which is consistent with the thickness we calculated for an adult *Apatosaurus* based on *Sauroposeidon*, above. Intervertebral cartilage would still be 10–15% of bone length in the proportionally shorter cervicodorsals. Averaged over the whole neck, in the adult cartilage probably contributed about 10–12% to the length of the neck.

Unfortunately, none of the slices provide us with as clear an image of the condyle-cotyle separation as at the C5/C6 joint in *Sauroposeidon*. But we can investigate which of the hypothetical models (Figure 14) the real vertebrae more closely approach by measuring the thickness of the cartilage gap not only at the deepest part of the cotyle but also at its margins. By analysing the full sequence of slices we can see that in slice 46 (Figure 15 part F), the lateral walls of condyle and cotyle are orthogonal to the plane of the section (so the cartilage gap is not artificially inflated by measuring its width on a slice that cuts it at an angle). In that slice, the separation between condyle and cotyle is about 3.5 mm. In slice 37 (Figure 15 part E), the uppermost margins of condyle and cotyle are orthogonal to the plane of slice, and the separation is about 4 mm. These results are consistent with each other, showing that the condyle was not displaced toward the margin of the cotyle. However, this radial thickness of cartilage at the rim of the condyle and cotyle is only about one third of the maximum anteroposterior thickness of the cartilage from the front of the condyle to the deepest part of the cotyle. This indicates that the condyle is not concentric with the cotyle – in fact, it is considerably less rounded, just as in *Sauroposeidon*. As more articulated sauropod vertebrae are scanned, it will be interesting to see if this geometry of the intervertebral joint is a convergent feature of *Apatosaurus* and *Sauroposeidon* or something common to most or all sauropods.

Slice 33 is of particular interest because it shows the condyle centred in the cotyle and the left zygapophyses in articulation (Figure 15 part D). Adjacent slices confirm that the left zygapophyses are in tight articulation over their entire length. Cartilage thickness between the zygapophyses is 1–2 mm. Unfortunately, the zygapophyses on the right are not preserved. The tight articulation of the left zygapophyses combined with the centring of the condyle of D2 in the cotyle of D1 indicates that this posture was achievable in life.

Using various landmarks we estimate that D1 is extended 31–36° relative to D2. This degree of extension is noteworthy; it is considerably more than the ∼6° of extension that Stevens & Parrish [13], [17] estimated between the cervical vertebrae of adult specimens of *Apatosaurus* and *Diplodocus*. The anterior dorsals have very large zygapophyseal facets that are not as far from the centre of rotation as they are in most of the cervical series, and these large, advantageously-positioned zygapophyses may have facilitated a greater range of motion than is found in the middle of the neck. This is consistent with the finding that most extant tetrapods raise and lower their heads by extending and flexing at the cervicodorsal junction, rather than bending in the middle of the neck [45], [46]. It also reinforces the argument that flexibility of the anterior dorsal vertebrae should considered when trying to estimate the range of motion of the head and neck [22].

#### Apatosaurus louisae CM 11339

**Figure 16:**
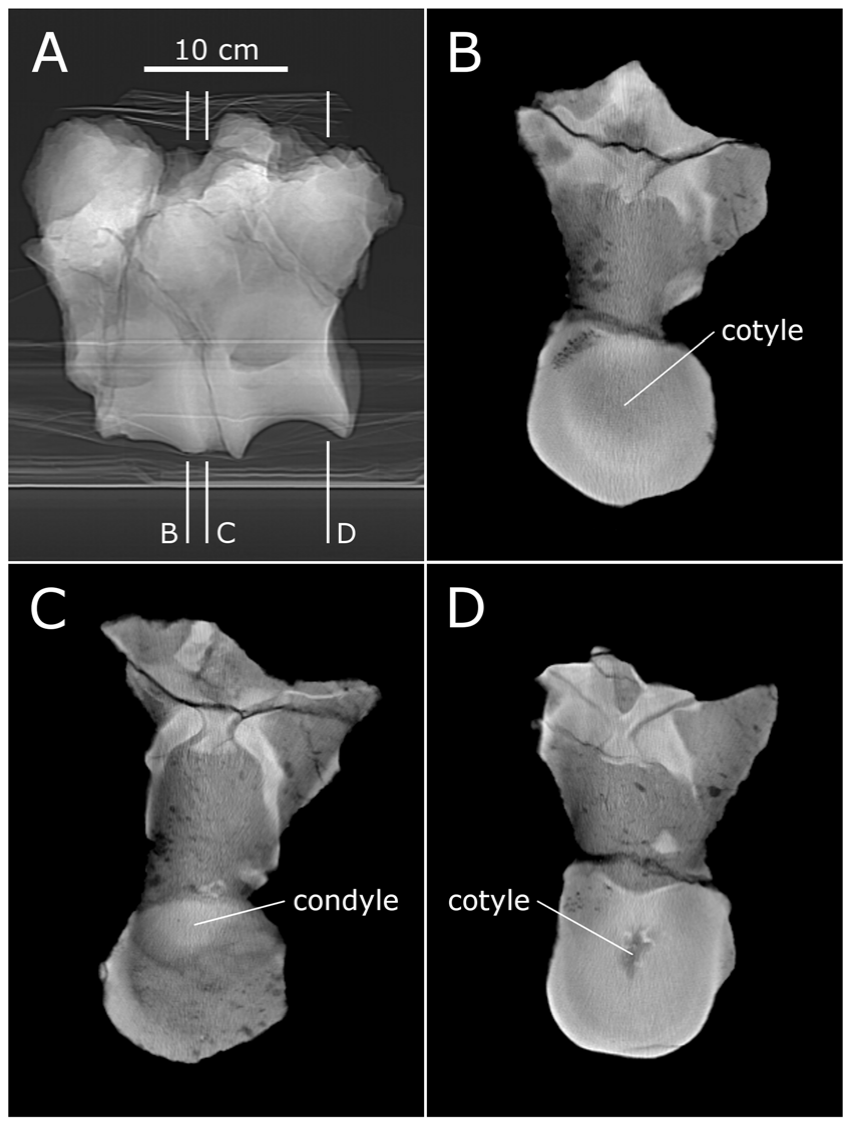
Dorsal vertebrae of *Apatosaurus* CM 11339. Articulated middle or posterior dorsal vertebrae of *Apatosaurus* CM 11339. A. X-ray scout image showing the two vertebrae in articulation, in left lateral view. B–D. Slices 39, 43 and and 70 in posterior view, showing the most anterior appearance of the condyles and cotyles.

CM 11339 includes a pair of articulated middle or posterior dorsal vertebrae, with hyposphene/hypantrum articulations (Figure 16). The more posterior of the two vertebrae has a cotyle height of 94 mm. Middle and posterior dorsal vertebrae of CM 3018 have cotyle heights of 315–365 mm, or 3.4–3.9 times the linear size of CM 11339. The individuals represented by CM 3399 and CM 11339 are therefore about the same size, roughly one quarter of the size of the large and presumably adult CM 3018. (They cannot however both represent the same individual as they contain overlapping elements – specifically, most of the dorsal column.)

The slice thickness in the CT scan is 5 mm, with 1.5 mm of overlap on either side, yielding a distance of 3.5 mm from the centre of one slice to the next. The cotyle of the anterior vertebra is first revealed in slice 39 (Figure 16 part B). The condyle of the second vertebra first appears in slice 43 (Figure 16 part C). The spacing between the vertebrae is therefore four slices (plus or minus one slice, as discussed above for CM 3390) or 14±3.5 mm. The first clear slice through the cotyle of the second vertebra is in slice 70 (Figure 16 part D). So the functional length of the second vertebra is 27±1 slices or 94.5±3.5 mm. The cartilage/bone ratio is therefore 14±3.5/94.5±3.5 or 15±4%.

### Data from turkey neck

Tables 1 and 2 contain all measurements made of the dissected turkey neck. The banner figures are as follows:

**Table 1:** Measurements of individual vertebrae of a turkey neck: anteroposterior lengths of centra and zygapophyseal facets, measured “wet” (freshly dissected), “dry” (after removal of all flesh and one day's drying) and “degreased” (after one day in dilute hydrogen peroxide and one week's thorough drying).

**WET**											
**Vertebra**	**Centrum**	**Prezyg**		**Postzyg**							
	**Length**	**L**	**R**	**L**	**R**						
A	22.5	6.78	7.3	7.86	8.48						
B	24.5	7.53	7.43	8.28	7.53						
C	25.05	7.43	6.76	7.63	8.87						
D	24.5	7.47	8.11	8.88	8.83						
E	24.5	8.45	8.86	8.96	9.27						
F	24	8.58	8.76	8.12	9.53						
G	22.8	9.28	9.51	8.46	9.67						
H	19.6	9.57	10.93	7.2	8.61						
Total/Avg	187.45	8.14	8.46	8.17	8.85						
		8.3		8.51							
**DRY**						**RATIO wet:dry**					
**Vertebra**	**Centrum**	**Prezyg**		**Postzyg**		**Vertebra**	**Centrum**	**Prezyg**		**Postzyg**	
	**Length**	**L**	**R**	**L**	**R**		**Length**	**L**	**R**	**L**	**R**
A	23.28	5.95	6.44	6.72	6.63	A	0.966	1.139	1.134	1.170	1.279
B	23.88	6.59	6.56	7.22	7.21	B	1.026	1.143	1.133	1.147	1.044
C	23.96	6.54	6.5	7.8	7.82	C	1.045	1.136	1.040	0.978	1.134
D	23.6	7.23	7.17	7.84	7.81	D	1.038	1.033	1.131	1.133	1.131
E	23.54	7.74	7.61	8.54	8.46	E	1.041	1.092	1.164	1.049	1.096
F	23.01	7.61	7.96	8.24	8.34	F	1.043	1.127	1.101	0.985	1.143
G	22.05	8.1	8.34	8.46	7.97	G	1.034	1.146	1.140	1.000	1.213
H	18.56	9.39	9.56	6.59	7.07	H	1.056	1.019	1.143	1.093	1.218
Total/Avg	181.88	7.39	7.52	7.68	7.66	Average	1.031	1.104	1.123	1.069	1.157
		7.46		7.67				1.114		1.113	
**DEGREASED**						**RATIO wet:degreased**					
**Vertebra**	**Centrum**	**Prezyg**		**Postzyg**		**Vertebra**	**Centrum**	**Prezyg**		**Postzyg**	
	**Length**	**L**	**R**	**L**	**R**		**Length**	**L**	**R**	**L**	**R**
A	23.15	5.89	6.5	6.42	7.84	A	0.972	1.151	1.123	1.224	1.082
B	23.72	6.6	6.52	7.17	7.43	B	1.033	1.141	1.140	1.155	1.013
C	23.8	6.39	6.37	7.67	7.54	C	1.053	1.163	1.061	0.995	1.176
D	23.56	6.93	7.06	8.25	7.69	D	1.040	1.078	1.149	1.076	1.148
E	23.52	7.83	7.55	8.55	8.39	E	1.042	1.079	1.174	1.048	1.105
F	22.96	7.48	7.95	8.18	7.98	F	1.045	1.147	1.102	0.993	1.194
G	22	8.08	7.56	7.78	7.58	G	1.036	1.149	1.258	1.087	1.276
H	18.52	10.1	9.7	8.01	7.17	H	1.058	0.948	1.127	0.899	1.201
Total/Avg	181.23	7.41	7.4	7.75	7.7	Average	1.035	1.107	1.142	1.060	1.149
		7.41		7.73				1.124		1.11	

All lengths in mm. This table is also available as file S1.

**Table 2:** Length measurements of a turkey neck.

Condition of neck	Length	Intact as
	(mm)	proportion
Intact before dissection	189.5	0.00%
Articulated sequence of wet vertebrae immediately after dissection	186	1.88%
Sum of lengths of individual wet centra	187.45	1.09%
Articulated sequence of vertebrae after removal of all flesh and drying	179	5.87%
Sum of lengths of individual dry centra	181.88	4.19%
Articulated sequence of vertebrae after degreasing in H2O2 and drying	178	6.46%
Sum of lengths of individual degreased centra	181.23	4.56%

For each measurement, the length of the intact neck is given as a proportion, indicating by what factor the various measurements would need to be increased to yield the true length in life.

The intact neck segment measured 189.5 mm from the most anterior to most posterior bone. Once the neck had been dissected apart into individual vertebrae, the length of the column of these vertebrae was 186.0 mm. After removing all cartilage and other soft tissue and drying the vertebrae, the articulated sequence shrank to 179.0 mm. And after degreasing in dilute hydrogen peroxide and fully drying, the same articulated column measured 178.0 mm. The intact neck, then, was 6.46% longer than the length derived from fully cleaned vertebrae whose condition would most closely approach that of fossilised vertebrae.

Therefore, in order to reconstruct the in-vivo length of any vertebra, it is necessary to add 6.46% to the length of the dry bone. The effect of this is shown in Figure 17. (For simplicity, we added the whole 6.46% to one of the articulating surfaces rather than adding 3.23% to each.) Although this illustration is only schematic, it gives a reasonable indication of the magnitude of the effect: measuring from the composite image, we find that the inclusion of articular cartilage increases intervertebral elevation by about 15° per joint. If this were replicated along a neck of 14 vertebrae, the resulting additional deflection of the anteriormost vertebra would be an enormous 210°.

An additional extension of 210° in neutral pose is plainly impractical as it would result in the head being carried upside-down and directed backwards. What this really shows is simply that necks are not habitually held in neutral posture [20].

The changes in measured zygapophyseal length were less consistent than those in centrum length, due to the difficulty of measuring the facets accurately: the limits of the facets are difficult to make out, especially when soft tissue is present. Although the general trend was for the measurements of any given facet to decrease as soft-tissue was removed, in a few cases the lengths measured for cleaned, degreased and dried zygapophyseal facets were longer than those taken from the vertebrae when freshly dissected. It seems unlikely that these measurements are correct: probably the earlier measurements underestimated the facet lengths. However, we have used the figures as measured rather than “fudging”, in the hope that any over- and under-measurements cancel out across the whole data set.

With these caveats, the key zygapophyseal measurements are that the average lengths of pre- and postzygapophyseal facets when freshly dissected (i.e., including cartilage) were 8.30 and 8.51 mm respectively; and that the corresponding lengths from cleaned, degreased and dried facets were 7.41 and 7.73 mm. This means that the additional length contributed by cartilage is 12% for prezygapophyses and 11% for postzygapophyses, an average of about 11%. Measurement error means that the true figure may be rather more than this (or conceivably slightly less), but we will use the figure 11%.

### Data from other animals

Turkeys are not the only animals whose intervertebral cartilage can shed light on that of sauropods. Some data are available for certain other animals, though not yet in as much detail as above. Note, however, that these data are only indicative, and cannot in general be compared directly with those above as they were obtained by a variety of different methods.

The cartilage of other birds is also informative, since all modern birds are equally closely related to sauropods. Of particular interest is the ostrich, as it is the largest extant bird. In a sequence of 14 cervical vertebrae (C3–C16) the total length of the centra when wet and with cartilage intact was 865.5 mm, but after drying and removal of cartilage only 814 mm [47]. Thus intervertebral cartilage accounted for an increase of 51.5 mm, or 6.3% over the length of bone alone.

**Figure 18:**
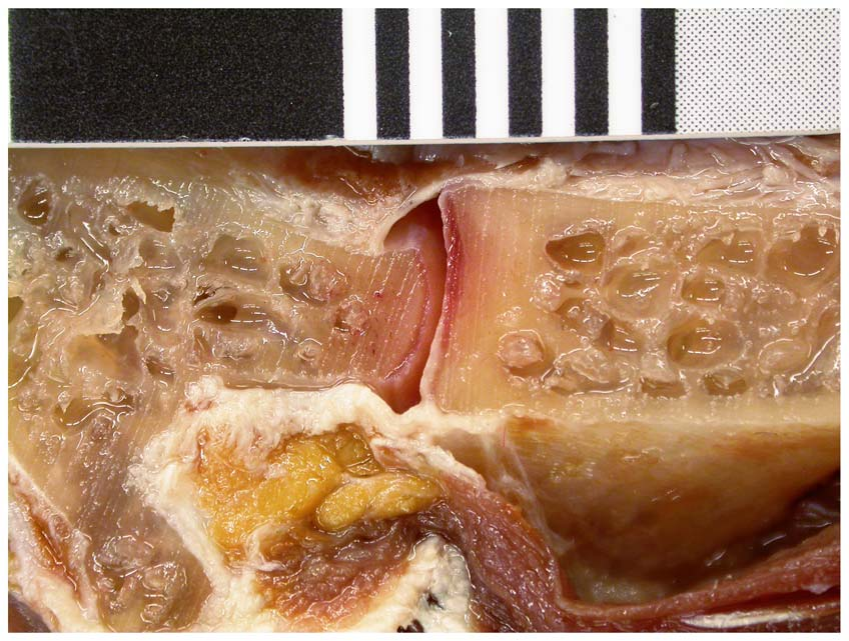
Cartilage in the neck of a rhea. Joint between cervicals 11 (left) and 10 (right) of a rhea, sagittally bisected. Left half of neck in medial view. The thin layers of cartilage lining the C11 condyle and C10 cotyle are clearly visible.

The rhea is closely related to the ostrich, but has very different intervertebral cartilage. Measuring the cartilage thickness on both sides of the vertebrae of a sagittally bisected rhea neck (Figure 18), we found that on average cartilage added 2.59% to the length of the vertebrae (Table 3).

**Table 3:** Measurements of centrum lengths and intervertebral cartilage in the sagittally bisected neck of a rhea.

	Segment length			Condyle cartilage			Cotyle cartilage			Bone	Cartilage%	
	Left	Right	Avg.	Left	Right	Avg.	Left	Right	Avg.	Length	Of bone	Of total
C4	32.3	31.9	32.1			0.43	0.51		0.51	31.2	3.00	2.91
C5	36.1	36.8	36.5	0.41		0.41	0.93		0.93	35.1	3.82	3.68
C6	39.3	39.2	39.3		0.57	0.57	0.58		0.58	38.1	3.02	2.93
C7	39.9	40.3	40.1			0.43	0.74	0.47	0.61	39.1	2.64	2.57
C8	41.5	41.1	41.3			0.43	0.44	0.39	0.42	40.5	2.08	2.03
C9	41.8	42.4	42.1	0.36		0.36	0.57	0.43	0.50	41.2	2.09	2.04
C10	40.6	41.0	40.8		0.42	0.42	0.53	0.43	0.48	39.9	2.26	2.21
C11	38.3	38.6	38.5	0.31	0.47	0.39	0.32	0.38	0.35	37.7	1.96	1.92
C12	37.4	37.0	37.2	0.39	0.43	0.41	0.40	0.35	0.38	36.4	2.16	2.11
C13	34.2	33.8	34.0	0.48	0.39	0.44	0.58	0.47	0.53	33.0	2.91	2.82
Avg.	38.14	38.21	38.2	0.39	0.46	0.42	0.56	0.42	0.53	37.2	2.59	2.52

All measurements are in mm. “Segment” here means a centrum including its anterior and posterior articular cartilage. Empty cells represent surfaces so torn up by the bandsaw used in bisection that accurate measurements were impossible. There are more of these empty cells on the right than on the left because of how the saw trended; the cut was not perfectly on the midline. For C4, C7 and C8, condyle cartilage thickness could not be accurately measured on either side, so an estimate of the average was used. This table is also available as file S2.

**Table 4:** Cervical intervertebral cartilage thickness in a variety of taxa, expressed as a percentage of bony centrum length.

Taxon	Thickness	Reference	Notes
*Sauroposeidon*	4.50%	This study	Measurements from CT scan of articulated material. Vertebrae are proportionally long mid-cervicals; averaged over the whole neck the thickness is estimated to have been 5.8%.
*Apatosaurus* CM 3390	16–24%	This study	Measurements from CT scan of articulated material. Vertebrae are most anterior dorsals.
*Apatosaurus* CM 11339	14.80%	This study	Measurements from CT scan of articulated material. Vertebrae are middle or posterior dorsals.
Turkey	4.56%	This study	Difference in measurements of intact neck and articulated sequence of cleaned, degreased and dried vertebrae.
Ostrich	6.30%	[47]	Difference in measurements of individual vertebrae with and without cartilage.
Rhea	2.59%	This study	Measurement of *in situ* cartilage in bisected neck.
Alligator	14.90%	This study	Measurement of *in situ* cartilage from photograph of cross section.
Horse	6.90%	This study	Measurement of *in situ* cartilage from photograph of cross section.
Camel	13.00%	This study	Crude measurement from condyle margin to cotyle lip of lateral-view X-ray. This is an interim measurement, which we hope to improve on when we obtain better images.
Dog	17.00%	This study	Measurement of intervertebral gaps in lateral-view X-ray, uncorrected for likely concavity of cotyles.
Giraffe	24.00%	This study	Difference in measurement of intact neck and closely articulated sequence of cleaned vertebrae. Young juvenile specimen.
*Muraenosaurus*	14.00%	[58]	Measurement of *in situ* cartilage in fossils.
*Cryptoclidus*	20.00%	[58]	Measurement of *in situ* cartilage in fossils.

**Figure 19:**
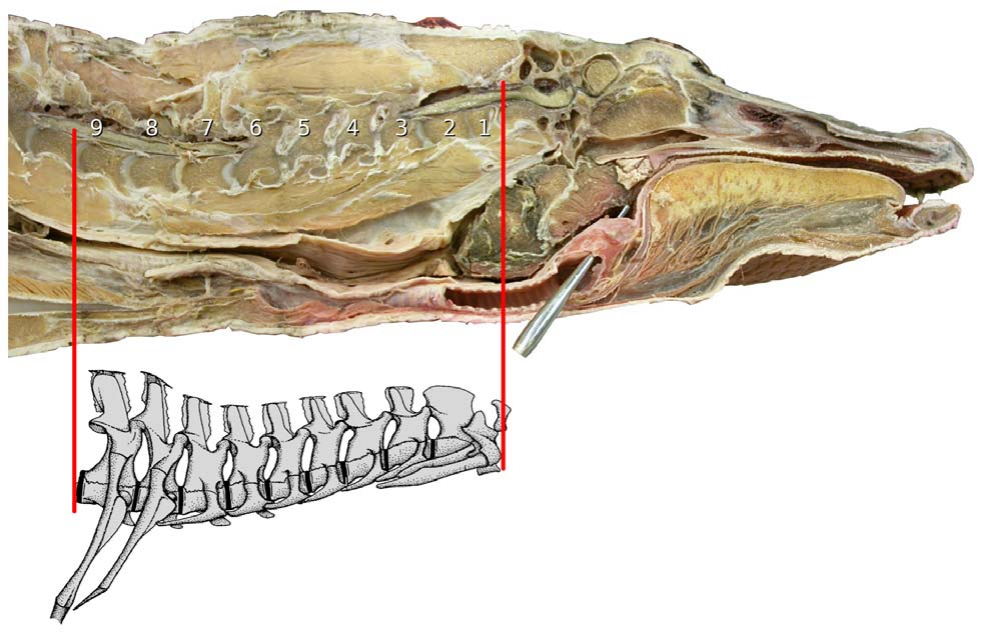
Alligator head and neck. Sagittally bisected head and neck of American alligator, with the nine cervical vertebrae indicated. Inset: schematic drawing of these nine vertebrae, from ([62]: figure 1), reversed.

Among extant animals, crocodilians are the next closest relatives to sauropods. Therefore, birds and crocodilians together form an extant phylogenetic bracket. We examined a sagittally bisected frozen American alligator. This animal was wild-caught and so its exact age is not known, but the snout-vent length of 51 cm suggests an age of about one year. We measured the thickness of intervertebral cartilage from photographs (Figure 19) using GIMP [48], a free image-editing program similar to PhotoShop. We found that of a total neck length of 779 pixels, 101 pixels were cartilage, constituting 14.9% of the length of the bone (678 pixels).

**Figure 20:**
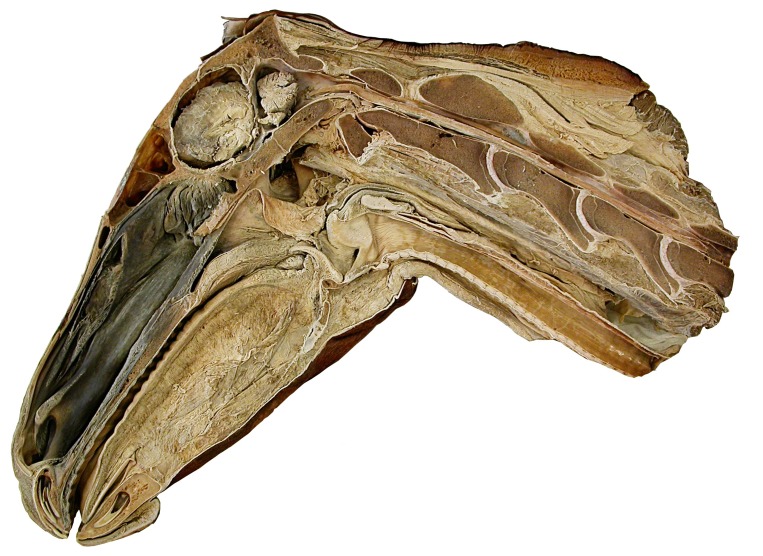
Horse head and neck. Sagittally bisected head and anterior neck of a horse. The first four cervical vertebrae are complete, but the posterior part of the fifth is absent. Note that the condyles are deeply embedded in their cotyles.

The horse is of interest as a good-sized animal with a reasonably long neck and strongly opisthocoelous cervical vertebrae – that is, having vertebrae with pronounced condyles and cotyles rather than flat articular surfaces. From photographs of a sagittally bisected horse head and neck (Figure 20), we measured the thickness of intervertebral cartilage for three vertebrae (C2, C3 and C4). C5 was broken and more posterior vertebrae were absent. Of a total C2–C4 neck length of 940 pixels, 61 pixels were cartilage, constituting 6.9% of bone length (879 pixels). This thickness of neck cartilage is consistent with those illustrated in veterinary radiographs [49]–[52].

**Figure 21:**
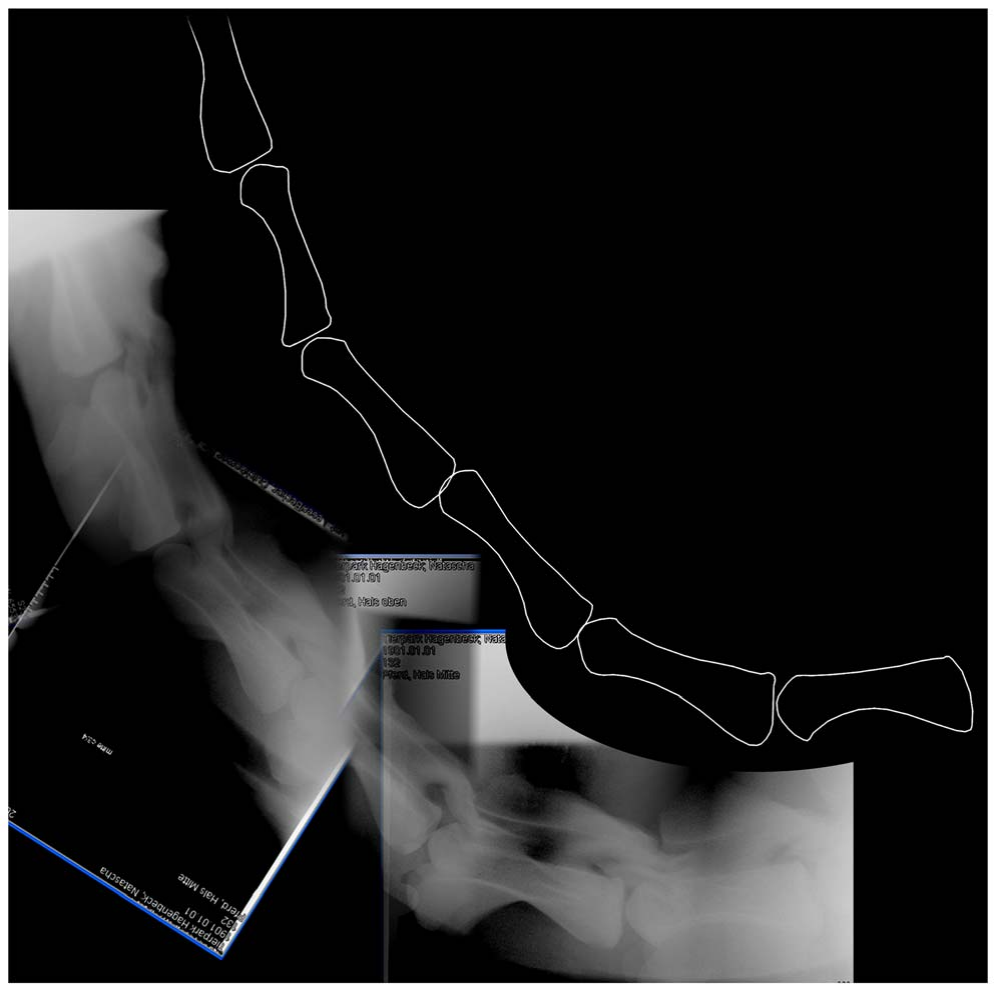
Camel neck in X-ray. X-ray image of a camel, with tracing to highlight the centra of cervical vertebrae 2–7. (C1 and the anterior part of C2 are obscured by the skull.) Note that most of the condyles do not even reach the posterior margins of their corresponding cotyles, let alone embed deeply within them.

Camels also have long necks and opisthocoelous cervical vertebrae. We might expect their necks to be similar to those of horses, but X-rays show that they are very different (Figure 21). While the condyles of horses' cervicals are deeply inserted into their corresponding cotyles, those of the camel do not even reach the posterior lip of their cotyles, so that a clear gap is visible between centra in lateral view. (The same is true in alpacas [53], [54].) It is difficult to measure the thickness of cartilage when much of it is hidden inside the cotyle; however, we were able to obtain a rough measurement of 13% the length of the bones, by measuring cartilage space from condyle rim to cotyle margin. The example of the camel contradicts Stevens and Parrish's claim, quoted in the introduction, that “the mammalian opisthocoelous biomechanical design [consists] of condyles that insert deeply in cotyles of matching curvature, leaving little room for cartilage […] vertebrae with nonplanar central articular geometry generally have little intervening cartilage (pers. obs.), and thus little room for conjecture regarding their undeflected state”. Instead, the situation is more complex: different animals have very different arrangements and the bones alone may not convey sufficient information.

**Figure 22:**
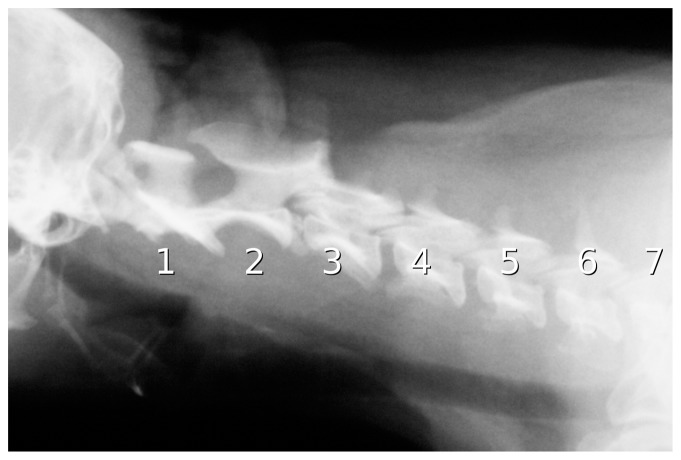
Dog neck in X-ray. Neck of a dog (dachsund), in X-ray, with the seven cervical vertebrae indicated. This photo has been used with permission from the Cuyahoga Falls Veterinary Clinic.

From a veterinary X-ray of a dog (*Canis familiaris*) we measured a total length from the posterior margin of C2 to that of C6 of 881 pixels (Figure 22). The intervertebral gaps behind the four vertebrae C2–C5 were 28, 34, 37 and 39 pixels, for a total of 138. This constitutes 18.6% of bone length (743 pixels). However, the true thickness of cartilage was probably greater, since the intervertebral gaps visible in lateral view are from the posterior margin of the cotyle to the anterior margin of the condyle. Allowing for the additional thickness of cartilage within the cotyles would add perhaps 1/4 to these measurements, bringing the cartilage proportion up to 23%. This neck X-ray is consistent with those of other dogs illustrated in the veterinary literature [55]–[57].

**Figure 23:**
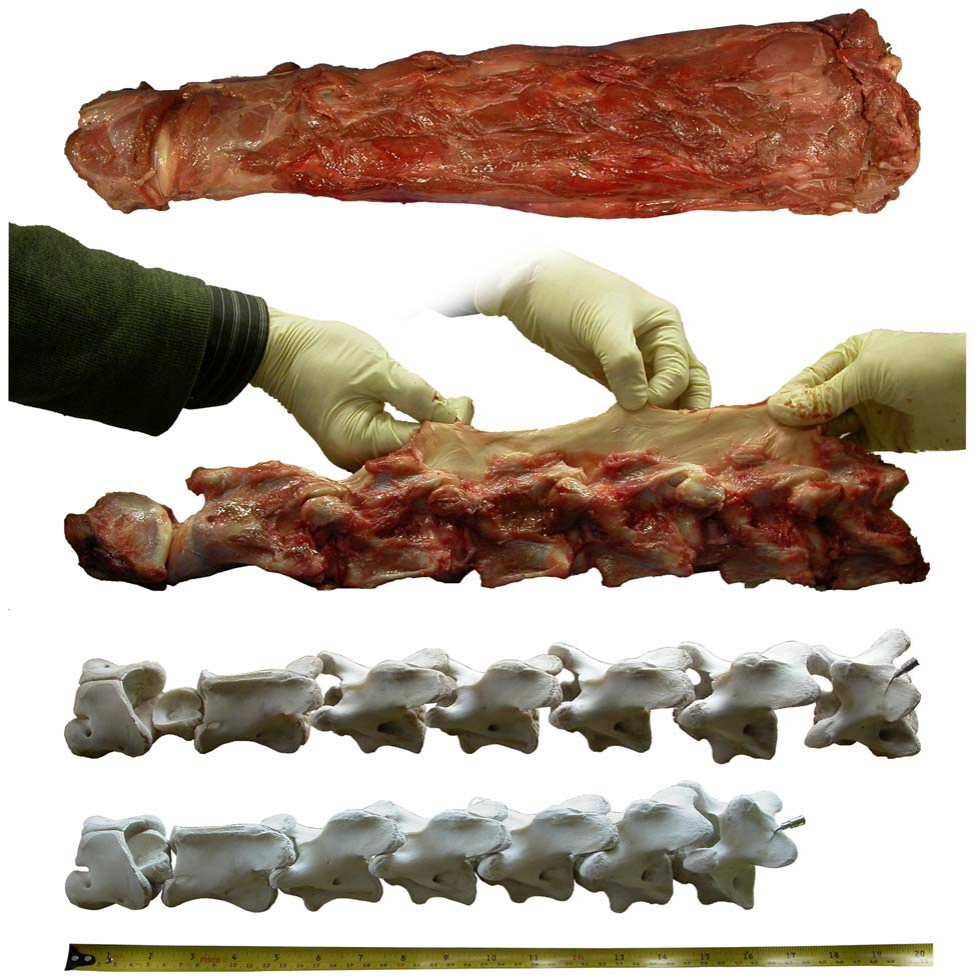
Neck of a young juvenile giraffe. Neck of a young juvenile giraffe, in various states of dissection, to scale. Top, the neck as received, skinned and stripped of skin, oesophagus and trachea. Second, the neck with most muscle removed and the nuchal ligament stretched out. Third, the vertebrae cleaned of soft tissue and cartilage, laid out with equal intervertebral spacing to attain the same total length as when intact (51 cm). Fourth, the vertebrae in the same condition but articulated as closely as possible, forming a misleading cervical skeleton measuring only 41 cm. Top image in left lateral view; second in right lateral view, reversed; third and fourth in left dorsolateral.

The best extant sauropod analogue would be the giraffe (*Giraffa camelopardalis*), due to its larger size and much longer neck. Unfortunately, giraffe necks are difficult to come by, and the only data we have been able to gather was from the neck of a young juvenile, two weeks old at the time of death. When intact, the neck was 51 cm in length; but when the vertebrae were prepared out and cleaned of cartilage, they articulated to form a misleading cervical skeleton that is only 41 cm long (Figure 23). In this neck, intervertebral cartilage contributes 24% of the length that the bones themselves contribute. No doubt this very high ratio is largely due to the incomplete ossification of the bones of a young juvenile: it would be interesting to carry out the same exercise with the neck of an adult giraffe, to see whether giraffes more closely resemble camels or horses in the thickness of their intervertebral cartilage.

Finally, Evans [58] measured the thickness of intervertebral cartilage preserved in the complete, articulated fossilised necks of two plesiosaurs. He found that it came to 14% of centrum length in *Muraenosaurus* and 20% in *Cryptoclidus*.

These results are summarised in Table 4. Across all 13 surveyed animals, and using midpoints of ranges for *Apatosaurus*, the mean cartilage/bone ratio is 12.5%, and the median is 14.0%. But there is a great deal of variation (standard deviation  = 6.9%). For this reason, and because some juvenile individuals were included, and because the measurements were obtained by a variety of different methods, simple averages are not reliable. With that caveat, averages by clade are as follows: sauropods 13.2%, birds 4.5%, crocodilians 14.9%, mammals 15.2% and plesiosaurus 17%.

## Discussion

### Implications for sauropod necks

The morphology of intervertebral cartilage in the sauropods is not known, and cannot presently be determined from osteological correlates, as none have yet been identified for bird- and mammal-style intervertebral joints. It is notable that in the examined extant animals with true intervertebral discs (crocodilians and mammals) the cartilage:bone ratios are three times higher than in birds. The relatively low cartilage ratio for *Sauroposeidon* and the high ratio for *Apatosaurus*, taken in isolation, perhaps suggests some variation in morphology within Sauropoda, with *Sauroposeidon* having bird-style synovial intervertebral joints and *Apatosaurus* having true discs. Such variation would not be unprecedented: the presence of simple articular discs in the ostrich and their absence in the rhea shows that variation exists even at low taxonomic levels. However, the difference in proportional cartilage thickness between these two sauropods is more parsimoniously explained as due to the elongation of the *Sauroposeidon* vertebrae and the juvenile nature of the *Apatosaurus* specimens.

As shown by the contrasting morphology of horse and camel necks, similarly shaped vertebrae of different animals may be augmented by a dramatically different shape and amount of cartilage. It may be that, in the same way, different sauropods had significantly different cartilaginous contributions to their necks. Given information regarding one sauropod group, we must be cautious not to assume that it generalises to all others.

With these caveats in mind, and based on the limited information currently available, it is reasonable to guess that most adult sauropods had cartilage/bone ratios of about 5–10% – that the lower figure for *Sauroposeidon* is a result of its extreme vertebral elongation and the higher figure for *Apatosaurus* is due to its proportionally shorter vertebrae. We obtained similar estimates for the cartilage thickness in an adult *Apatosaurus* neck by scaling up from the juvenile material and scaling down, proportionally, from *Sauroposeidon*, which suggests that unlike mammals, juvenile sauropods may not have had proportionally thicker intervertebral cartilage than adults.

In the neck of a turkey, adding 4.56% to bony centrum length to restore the absent cartilage resulted in neutral pose being raised by 15° at each joint. This increase in extension is roughly proportional to the proportion of cartilage restored and inversely proportional to the height of the zygapophyses above the centre of rotation – very high zygapophyses would mean that the increased length of the centrum with cartilage restored would subtend only a small angle at the zygapophyses, while low zygapophyses would result in a wider angle. Zygapophysis height varies among different sauropods, and along the neck of each; but as a proportion of centrum length it is generally reasonably close to that of turkey cervicals. It therefore seems reasonable to conclude that restoring the missing cartilage to sauropod vertebrae would raise neutral posture commensurately, although it is not possible to give meaningful quantitative results without detailed modelling.

If the neutral posture of each joint in a sauropod's neck was raised, perhaps by as much as 15°, it may seem that this would result in an absurd neutral posture in which the neck curls back over the torso. In practice, as has often been noted [20], [45], [46], animals do not hold their necks in neutral posture, but habitually extend the base of the neck and flex the more anterior portion. This pattern of behaviour combined with more extended neutral postures than previously envisaged indicates that swan-like postures may have been very common, and that in some sauropods it may have been common to hold the middle region of the neck at or even beyond vertical.

We found that the anteroposterior length of the zygapophyseal facets of turkey cervicals were, on average, 11% longer when cartilage was intact than after it was removed. It is reasonable to assume that a similar proportion held for sauropods. The effect of longer zygapophyseal facets on ROM is very straightforward: ROM increases more or less linearly with zygapophyses length, so an 11% increase in the latter translates directly to an 11% increase in dorsoventral flexibility at each neck joint. Of course, if the neck were thought for other reasons to be very inflexible, an 11% increase in small ROM angles would not make a particularly big difference. Calculating absolute values for ROM requires detailed modelling that is beyond the scope of this study.

In apparent contradiction to this, recent work [47] shows that ostrich necks with their soft tissue in place are less flexible than bones alone indicate, and suggests that the same would have been true of sauropod necks. In interpreting this result, it is important to bear two things in mind. First, whatever it may do to range of motion, including intervertebral cartilage unquestionably raises neutral pose: it is for this reason that the habitual life posture of rabbits is more raised than can be attained by the bones of the neck even in maximum extension [20]. Second, the effect of soft tissues on neck flexibility differs among taxa. For example, in humans, where the cervical vertebrae are mildly amphicoelous, there is no ball-and-socket joint, so no obvious way for one vertebra to rotate with respect to those before and after it. But the thick intervertebral discs, with their roughly spherical nuclei, provide a centre of rotation: as the neck flexes and extends, the discs become wedge-shaped to accommodate motions that the bones alone would not permit [59]. More comparative work is needed to determine the different effects of soft tissue on flexibility in different taxa, and to enable conclusions to be drawn regarding extinct animals.

In summary, including cartilage in our models of sauropod necks shows that they were longer, more raised and probably more flexible than previously recognised.

### Future work

This study represents only a beginning, not an end, to the work on the neck cartilage of sauropods (and other extinct animals). We would like to see future work extend this in the following ways.

- CT scans of more sauropod neck segments that preserve vertebrae in articulation – ideally much more complete necks than the ones described here.
- Measurements of intervertebral cartilage thickness and zygapophyseal cartilage extent for more extant animals: especially birds and crocodilians, which together form an extant phylogenetic bracket for sauropods; and an adult giraffe, which has much the longest neck of any extant animal.
- Intervertebral and zygapophyseal cartilage measurements for individuals of different growth stages within single species, to determine how the amount and shape of cartilage varies through ontogeny.
- Work to determine whether dry bones have any osteological correlates that are informative regarding the morphology of intervertebral cartilage: true intervertebral discs, or synovial joints with or without articular discs.
- Finally, we would very much like to see the results of re-running the DinoMorph software with its models updated to take into account intervertebral and zygapophyseal cartilage. At present this is the only software that has been used to model intervertebral joints; if it remains unavailable then it may be possible to use more general-purpose CAD packages to achieve the same ends.

## Conclusions

A survey of intervertebral spacing and cartilage thickness in extinct and extant amniotes reveals several factors that affect any attempts to model vertebral articulations:

1. The thickness of intervertebral cartilage is highly variable among taxa, ranging from 2.6% of centrum length in a rhea to 24% of centrum length in a baby giraffe. Even if we restrict the sample to presumably adult animals, the range is 2.6% to 20% – a factor of almost eight.
2. There seem to be some systematic differences among clades: mammals and other non-avian amniotes typically have thicker intervertebral cartilage than birds. Intervertebral spacing is particularly high in plesiosaurs, perhaps because of their proportionally short vertebral centra (i.e., the cartilage was not thicker absolutely than in similarly sized animals, but only in comparison to the shorter vertebrae).
3. Based on our admittedly limited sample, sauropods appear to have been intermediate between birds and other amniotes in the thickness of the intervertebral cartilage in the neck, with cartilage accounting for 5–10% of the lengths of the centra in adults.
4. Although only two of our sampled sauropod specimens have strongly opisthocoelous centra, in both of those cases the bony condyle is not shaped to fit the cotyle, and the intervertebral cartilage is thicker anteroposteriorly at the centre than radially at the cotyle rim. More sampling is required to determine if this is a general feature of sauropods, convergent in *Apatosaurus* and *Sauroposeidon*, or variable among individuals and along the column.
5. At present, there are no known osteological correlates of different intervertebral joint types (intervertebral disc, synovial joint, synovial joint with articular disc).
6. At present, there are no known osteological correlates of thick versus thin intervertebral cartilage. For example, horses and camels both have strongly opisthocoelous cervical vertebrae, but their intervertebral spacing is very different: in camels, the condyles do not even reach the rims of the cotyles, much less articulate with them directly.

These difficulties and uncertainties do not render attempts to model intervertebral joint mechanics uninformative or worthless. However, it is clear that intervertebral cartilage is a significant fraction of the length of the bony cervical series in most amniotes, as well as highly variable among taxa. Therefore, assumptions about intervertebral cartilage in biomechanical models must be explicit in choice of reference taxa, type of intervertebral joint, and thickness of cartilage. Sensitivity analyses using DinoMorph or other CAD software to quantify the variation in ONP and ROM imposed by different starting assumptions would be extremely valuable; indeed it is difficult to see how digital ONP and ROM estimates can be useful in the absence of such analyses. Recent work on the prosauropod *Plateosaurus* [28], [60] shows how this can be done for extinct dinosaurs; applying these techniques to sauropod necks would be informative.

More generally, we need to look more carefully at both fossils and extant organisms. In the extant realm, a search for possible osteological correlates of intervertebral joint type and cartilage thickness is very badly needed. But aside from that, simply documenting the cartilage thickness in a wider range of taxa will be useful in elucidating ontogenetic, phylogenetic, and size-related variation among individuals and clades. The same survey can be extended to articulated fossil material. Although complete, undistorted cervical material is rare for sauropods, a more extensive and careful survey of articular morphology will allow future workers to better constrain their models, and may also turn up characters of potential biomechanical and phylogenetic interest, such as the unusually flattened condyles in middle cervical vertebrae of *Sauroposeidon*. All specimens that have both centra and zygapophyses in articulation should be CT scanned where this is logistically feasible.

We have attempted a first step toward understanding how intervertebral cartilage affected the postures and ranges of motion of sauropod necks. We hope that further work makes this paper obsolete very quickly.

## Supporting Information

File S1 **Data from** **Table 1** **in more useful format.**
(XLS)

File S2 **Detailed data on rhea neck cartilage.**
(XLS)
